# Defect structure of yttria-stabilized hafnia nanoparticles

**DOI:** 10.1107/S2052252526003829

**Published:** 2026-06-09

**Authors:** Magnus Nørgaard Kløve, Andreas Dueholm Bertelsen, Mads Ry Vogel Jørgensen, Bo Brummerstedt Iversen

**Affiliations:** a Center for Sustainable Energy Materials Department of Chemistry Aarhus University Langelandsgade 130 8000 Aarhus C Denmark; b MAX IV Laboratory Lund University Fotongatan 224 84 Lund Sweden; ESRF, France

**Keywords:** yttria-stabilized hafnia, defect structure, *in situ*X-ray scattering, solvothermal synthesis, crystal structure

## Abstract

High-tem­per­a­ture polymorphs of hafnia (HfO_2_) are of significant inter­est in electronics and fuel-cell applications, and stabilization at ambient conditions can be achieved by aliovalent substitution and nanosize effects. Y^3+^ stabilization of hafnia (YSH) introduces local cation disorder around charge-com­pensating oxygen-ion vacancies, and here we establish both the average and local structure of YSH nanoparticles using synchrotron powder X-ray diffraction (PXRD) and pair distribution function (PDF) analysis. A range of phase-pure crystalline nanoparticles of Hf_1–*x*_Y_*x*_O_2–*x*/2_ were prepared *via* continuous flow solvothermal synthesis and subsequent high-tem­per­a­ture annealing, and full stabilization of the cubic phase is achieved already at 13 at% Y^3+^. The average structure conforms to the cubic fluorite phase of HfO_2_, but local displacive disorder caused by electrostatic attraction of neighbouring oxygen ions and repulsion of neighbouring metal ions by the net-positive oxygen-ion vacancies is established. The well-known Zr_3_Y_4_O_12_ structure, which incorporates such relaxation motifs, provides a good pr­oxy description of YSH. *In situ*X-ray total scattering experiments provide insight into the formation mechanism of the YSH nanoparticles and initial precipitation of an atomically mixed amorphous phase is followed by crystallization over several minutes. The crystallization rate in­creases with higher reaction tem­per­a­ture, whereas an increased doping level results in slower crystallization.

## Abstract 2

Local disorder in nanoparticles of yttria-stabilized hafnia (YSH) is identified by combined powder X-ray diffraction (PXRD) and pair distribution function (PDF) analysis. The aliovalent doping with Y^3+^ introduces oxygen vacancies that electrostatically distort the local coordination of the neighbouring ions, thus resulting in both vacancy disorder, mass disorder and displacive disorder in these otherwise well-crystalline YSH nanoparticles.

## 1. Introduction

Hafnia (HfO_2_) is a rather ubiquitous transition-metal oxide, attracting increased attention, especially in the field of metal-oxide semiconductor field-effect transistors (MOSFETs). With a high dielectric constant and chemical com­patibility with Si, HfO_2_ is a potential candidate to replace SiO_2_ as gate dielectrics in gate stacks of MOSFETs to prevent current leakage when decreasing transistor size (Robertson, 2004 ▸; Robertson & Wallace, 2015 ▸). The first discovery of ferroelectricity in Si-doped HfO_2_ thin films in 2011 (Böscke *et al.*, 2011 ▸), and later in Y-doped HfO_2_ thin films (Müller *et al.*, 2011 ▸), sparked hopes of leveraging the ferroelectric behaviour under MOSFET read operation. If doped, HfO_2_ also exhibits oxygen-ion con­duc­tivity and thus has potential use as an electrolyte in solid oxide fuel cells or electrolysers (Weyl & Janke, 1996 ▸). The dielectric behaviour and oxygen-ion con­duc­tivity in HfO_2_, how­ever, rely greatly on the ability to stabilize its high-tem­per­a­ture polymorphs (Zhao & Vanderbilt, 2002 ▸; Wang *et al.*, 2012 ▸; Fischer & Kersch, 2008 ▸; Alotaibi *et al.*, 2021 ▸). Because of very low thermal con­duc­tivity, HfO_2_ also sees use in thermal barrier coatings in high-tem­per­a­ture applications, such as metal protection in gas turbine engines (Lakiza *et al.*, 2021 ▸; Wu *et al.*, 2023 ▸).

Like its Group IV neighbour ZrO_2_, HfO_2_ adopts a monoclinic structure (*P*2_1_/*c*, *m*-HfO_2_) at ambient conditions. At ∼2000 K, it transforms into a tetra­gonal structure (*P*4_2_/*nmc*, *t*-HfO_2_) that eventually transforms into the cubic fluorite structure (*Fm*
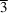
*m*, *c*-HfO_2_) at ∼2800 K (Cardarelli, 2008 ▸; Shin *et al.*, 2006 ▸). The phase transformations are martensitic in nature and the structures involve only slight reorganization upon transformation (Tang *et al.*, 2005 ▸). Although the coordination number (CN) of Hf^4+^ changes from seven in *m*-HfO_2_ to eight in both *t*-HfO_2_ and *c*-HfO_2_, the three structures share a great resemblance, as evident from a com­parison of the crystal structures (Fig. S1 in the supporting information). From powder X-ray diffraction (PXRD), the two high-tem­per­a­ture polymorphs are difficult to distinguish if any significant instrumental or size broadening effects are present in the data (Fig. S2). The small peak splitting, visible as a faint asymmetry, *e.g.* in the second Bragg peak, is the only significant deviation between the two structures.

The high-tem­per­a­ture polymorphs are suggested to be stable in nanocrystalline powders because of their lower surface enthalpy com­pared to *m*-HfO_2_, with ‘critical sizes’ for *c*/*t*-HfO_2_ to *m*-HfO_2_ crossings in the range 30–40 nm (Sharma *et al.*, 2018 ▸; Zhou *et al.*, 2012 ▸). A similar rationale was originally proposed for ZrO_2_ (Garvie, 1965 ▸), but the literature is scarce on size stabilization of undoped HfO_2_ (Lu *et al.*, 2008 ▸; Kumar *et al.*, 2017 ▸; Wan & Zhou, 2017 ▸). In fact, several studies indicate a more com­plex behaviour, where *m*-HfO_2_ is stable significantly below the suggested ‘critical size’ limits (Lu *et al.*, 2008 ▸; Christensen *et al.*, 2021 ▸; Waetzig *et al.*, 2016 ▸; Chaubey *et al.*, 2012 ▸).

Aliovalent doping with di- and trivalent cations, such as Group II, Group III and lanthanide cations, presents another avenue for stabilization of the high-tem­per­a­ture polymorphs in both HfO_2_ and ZrO_2_ (Kim, 1989 ▸). As a charge-com­pen­sa­ting mechanism, aliovalent doping generates oxygen vacan­cies, and it is believed that these vacancies stabilize the high-tem­per­a­ture phases by effectively giving a CN of seven for Zr^4+^/Hf^4+^, as in the monoclinic polymorph (Lee *et al.*, 2008 ▸; Schmidt *et al.*, 2023 ▸; Li *et al.*, 1994*a* ▸). Y^3+^ doping of ZrO_2_, *i.e.* yttria-stabilized zirconia (YSZ), is the archetypical example of fluorite structure stabilization and the com­pound is widely used industrially as a solid oxide-ion electrolyte (Singh *et al.*, 2021 ▸). The hafnium analogue, *i.e.* yttria-stabilized hafnia (YSH), is less studied, but it displays a similar ability to stabilize the high-tem­per­a­ture phases and show ion con­duc­tivity, albeit slightly lower con­duc­tivity than that of YSZ (Sévin *et al.*, 2020 ▸; Zhang *et al.*, 2013 ▸; Trubelja & Stubican, 1991 ▸). Aliovalent Y^3+^ doping also has a profound impact on the thermal con­duc­tivity of HfO_2_ (Ramana *et al.*, 2012 ▸), since the mass disorder from Y^3+^ substitution on Hf^4+^ sites and the resulting oxygen-ion vacancies cause defect-phonon scattering, thus lowering the con­duc­tivity (Winter & Clarke, 2006 ▸; Klemens & Gell, 1998 ▸). Unlike YSZ, there is no consensus on the critical doping level for the com­plete stabilization of YSH, but both Matović *et al.* (2012 ▸) and Li *et al.* (2018 ▸) reported com­plete stabilization at 20 at% Y for microcrystalline powders. By extrapolation, Li *et al.* (2018 ▸) suggested a lower limit to be roughly 17 at% Y in com­parison with 14.8 at% Y (8 mol% Y_2_O_3_) in YSZ (Badwal, 1992 ▸).

By combining the two modes of stabilization, nanoparticles of YSH present an elegant way of stabilizing the high-tem­per­a­ture polymorphs at even lower Y^3+^ content. Upon coprecipitation of Hf^4+^ and Y^3+^ precursors using NH_4_OH, and subsequent annealing at 700 and 800 °C, com­plete stabilization at 10 at% Y for 10 and 12 nm YSH nanoparticles has been reported (Zhou *et al.*, 2012 ▸). Ball-milling and subsequent annealing at 1100 °C yielded com­pletely stabilized 58 nm YSH nanoparticles at a 10 at% Y doping (Qiu *et al.*, 2021 ▸). Other ways of synthesizing YSH nanoparticles include auto-com­bus­tion and subsequent annealing (Matović *et al.*, 2012 ▸) and sol–gel synthesis (Gálvez-Barbosa *et al.*, 2024 ▸).

Here, we report a novel approach to ensure efficient mixing of the constituent elements *via* continuous flow solvothermal synthesis using supercritical ethanol as solvent. High-tem­per­a­ture annealing of the amorphous Hf_1–*x*_Y_*x*_O_2–*x*/2_ powders induces crystallization and confirms the homogeneity of the as-prepared powders, as seen from the Vegard-like behaviour of the unit-cell parameters in accurate PXRD analysis, and corroborates that, apart from vacancies generated by alio­valent doping, additional stabilizing effects are responsible for lowering the critical doping limit at the nanoscale. While the long-range order of the YSH powders conform to the cubic fluorite phase of HfO_2_, anomalously high refined atomic displacement parameters (ADPs) and detailed local structure analysis *via* the atomic pair distribution function (PDF) reveal distinct local disorder in the YSH nanoparticles upon doping.

## 2. Experimental

### 2.1. Precursor preparation

Hafnium(IV) chloride (98%, Thermo Scientific Chemicals), yttrium(III) chloride (99.9%, ChemPur) and absolute ethanol (≥99.8%, VWR) were used as received. The precursor powders of Y^3+^ and Hf^4+^ were mixed in different ratios, giving a com­position series of Hf_1–*x*_Y_*x*_O_2–*x*/2_, and dissolved in ethanol under stirring for approximately 30 min. The total metal ion concentration was 0.1 *M* for all *ex situ* syntheses, whereas the concentration was 0.5 *M* for *in situ* experiments to increase the sample-to-background ratio. For the *ex situ* syntheses, samples with *x* = 0.08, 0.16, 0.32 and 0.48 were prepared, and for the *in situ* experiments, samples with *x* = 0.00, 0.08, 0.16, 0.24 and 0.32 were prepared.

### 2.2. *Ex situ* syntheses

Amorphous YSH powders were synthesized on a custom-built continuous flow reactor, employing a T-shaped mixing piece for the mixing of a pre-heated solvent stream of absolute ethanol and a stream of room-tem­per­a­ture precursor solution, prepared as described above. Additional details on the flow reactor can be found elsewhere (Hellstern *et al.*, 2015 ▸). The pressure inside the reactor was maintained at approximately 250 bar (1 bar = 10^5^ Pa) by adjusting a proportional-release valve. The flow rates were 4.67 and 4.00 ml min^−1^ for the solvent and precursor stream, respectively. With a synthesis tem­per­a­ture of 300 °C, a total flow rate of 8.67 ml min^−1^ through a reactor volume of 11.8 ml gives an approximate residence time of 39 s using equation (1),[Disp-formula fd1]
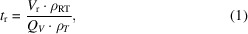
where *V*_r_ is the reactor volume, *Q_V_* is the total volume flow rate, ρ_RT_ is the room-tem­per­a­ture density of ethanol at 250 bar and ρ_*T*_ is density of ethanol at 250 bar and the desired synthesis tem­per­a­ture. Densities of ethanol at elevated tem­per­a­tures were obtained from the *REFPROP* software package (Huber *et al.*, 2022 ▸). After synthesis, the as-prepared powder suspensions were centrifuged, deca­nted and redispersed three times in H_2_O, ethanol absolute and then H_2_O again.

Powders were annealed at three different tem­per­a­tures (600, 900 and 1200 °C) in atmospheric air, were placed in an Al_2_O_3_ container and inserted in a preheated muffle furnace (Thermolyne Furnace, Thermo Scientific). The powders were in all cases removed after 3 h and left to cool at ambient tem­per­a­ture.

### 2.3. X-ray fluorescence spectroscopy

X-ray fluorescence (XRF) measurements were performed on a Rigaku NEX CG energy dispersive XRF analyzer to qu­antify the Y^3+^ content of the as-synthesized *ex situ* samples and to detect potential impurities. X-rays were generated from Al, Mo, Cu and RX9 targets. All measurements were performed under an He atmosphere. Qu­anti­fication was done with the built-in software, and spectra for all samples are shown in Figs. S3–S6 in the supporting information. Five separate measurements were performed on each powder sample, and the average of these and the standard deviation is reported.

Peaks from Hf, Y, Zr, Cl, Fe, Cr, Ni, Mn and Co were identified. A background measurement of an empty sample holder suggests that the instrument itself produces X-ray fluorescence for Zr, and as such, any qu­anti­fication of Zr is unreliable and has not been included in the qu­anti­fication. The qu­anti­fied amounts of the other elements are given in Table S1. The amount of Fe, Cr, Ni, Mn and Co corresponds to less than 0.5 mol% and presumably originates from corrosion of the stainless steel reactor tube. The detected amount of chloride is expected to originate from inadequate washing of the synthesized powders that bind the surface of the particles. Upon annealing, the chloride signal gradually diminishes and com­pletely vanishes after annealing at 1200 °C [Fig. S7(*a*)], thus suggesting chloride ions only bind to the surface of the particles and do not incorporate into the crystalline structure.

### 2.4. Scanning transmission electron microscopy

Scanning transmission electron microscopy (STEM) and energy-dispersive X-ray spectroscopy (EDS) were performed on an FEI Talos F200X instrument operated at 200 kV. A suspension of the as-prepared samples, as well as annealed samples, in ethanol (1 g powder in 1 ml ethanol) was first sonicated for 5 min and drop-casted onto TEM grids [Ultra-thin carbon (3 nm) on Lacey-Carbon–Copper mesh 400 from Plano GmbH]. Images were taken using a camera length of 98 mm, convergence angle of 10.5 mrad, a spot size of 5 and a high-angle annular dark field detector with a 60–200 mrad collection angle, together with the CemiSTEM X-ray detection system. The bright-field STEM images were collected using a 0–20 mrad collection angle. To improve statistics, an average of 14 pixels were binned in elemental maps.

### 2.5. Inductively coupled plasma optical emission spectroscopy

Inductively coupled plasma optical emission spectroscopy (ICP–OES) measurements of the atomic contents of the produced powders were performed on a Spectro ARCOS ICP–OES equipped with a Burgener Nebulizer and a Cyclo­nic Spray Chamber with an ASX-520 Autosampler. Standard series for Hf, Zr and Y were made from elemental standards from PlasmaCAL. The standard solutions were diluted to yield solutions for the measurements in the standard series with concentrations of 200, 150, 100, 10 and 1 ppm for Hf, and 50, 25, 10, 5 and 1 ppm for Zr and Y. The as-prepared YSH powders (∼20 mg) were dissolved in concentrated H_2_SO_4_ (1 ml) heated to 170 °C under stirring, and the solutions were then diluted to a volume of 25.00 ml with 1 *v*/*v*% HNO_3_. For determining the Hf and Y contents, these solutions were diluted additionally by a factor of four.

### 2.6. *In situ* experiments

The *in situ* experiments were con­ducted using a batch-type reactor cell made for *in situ*X-ray scattering that mimics the reaction conditions of the solvothermal flow reactor. The setup is described in detail elsewhere (Roelsgaard *et al.*, 2023 ▸; Becker *et al.*, 2010 ▸). The reactor cell consists of fused-silica capillaries (0.7 mm ID) coated with Kapton on the outside. The capillaries are pressurized using an HPLC pump (LabAlliance PrepPump) and heated using a jet of hot air calibrated to provide the desired reaction tem­per­a­ture inside the capillary. The desired tem­per­a­ture can be reached within a few seconds after heating (Roelsgaard *et al.*, 2023 ▸), thus mimicking the tem­per­a­ture gradient inside a continuous flow reactor. Contrary to the continuous flow reactor, the precursor probed by the X-ray beam is not under continuous flow, and so the *in situ* experiments investigate the reaction as a function of residence time. During the synthesis, X-ray scattering data are continuously collected. Nine *in situ* experiments were con­ducted, covering a com­position series (*x* = 0.00, 0.08, 0.16, 0.24 and 0.32) synthesized at 300 °C and a tem­per­a­ture series (200, 250, 300, 350 and 400 °C) for a com­position of *x* = 0.24.

### 2.7. X-ray total scattering

X-ray total scattering (TS) data measured on *ex situ* samples were collected at the DanMAX beamline at MAX IV (Lund, Sweden) with a photon energy of ∼35 keV (λ = 0.35537 Å). The scattering data were collected on a DECTRIS PILATUS3 X 2M CdTe 2D detector placed perpendicular to the X-ray beam path. Both data suitable for pair distribution function (PDF) and powder X-ray diffraction (PXRD) analysis were collected with sample-to-detector distances (SDDs) of ∼9 and ∼39 cm, respectively, to yield *Q*_max,inst_ of 20.8 and 11.3 Å^−1^. The powders were packed in 0.2 mm glass capillaries.

The *in situ* TS experiments were con­ducted at the P21.1 beamline at PETRA III (DESY, Hamburg, Germany), with a photon energy of 101 keV (λ = 0.12273 Å). For the com­position series, except *x* = 0.24, the scattering data were collected on a Perkin Elmer XRD 1621 2D detector, whereas the data for the tem­per­a­ture series were collected on a DECTRIS PILATUS3 X 2M CdTe 2D detector. The SDDs were 41.5 and 34.4 cm, resulting in a *Q*_max,inst_ of 21.8 and 25.0 Å^−1^, respectively.

All 2D detector images were azimuthally integrated using *PyFAI* (Kieffer & Karkoulis, 2013 ▸) after calibration of detector position and orientation based on a measurement of an Si NIST SRM 640d or LaB6 NIST SRM 660b.

### 2.8. Analysis of X-ray scattering data

The scattering data were analysed both in reciprocal space *via* Rietveld refinements of the PXRD data and in direct space by first obtaining the PDF and then analysing the data *via* a direct-space equivalent of Rietveld refinements.

The PDFs of *ex situ* samples were obtained using *GudrunX* (Soper & Barney, 2011 ▸). *GudrunX* handles incoherent scattering contributions by scaled table values by applying the method of Krogh-Moe (1956 ▸) and Norman (1957 ▸). Total scattering data obtained from an empty capillary and an empty beamline were used as background subtraction to remove non-sample coherent scattering contributions. The scattering patterns were corrected for a 2θ zero point offset determined from Rietveld refinement on an Si NIST SRM 640d standard measurement. The com­position from ICP–OES (Table 1 ▸) was used as the elemental com­position needed to obtain the PDFs, ignoring any contribution from Zr. For *ex situ* samples, a *Q*_min_ of 1 Å^−1^ and a *Q*_max_ of 20 Å^−1^ were used for the Fourier transform.

The PDFs from the *in situ* experiments were obtained using *PDFgetX3* (Juhás *et al.*, 2013 ▸), since this algorithm more efficiently treats large datasets. Here, the incoherent scattering contributions are treated using the *ad hocR*_poly_ polynomial correction to subtract the slowly varying incoherent features from the scattering patterns. *R*_poly_ was chosen to be 0.9 Å for all datasets, meaning that the PDF will only be affected by the correction below 0.9 Å (Juhás *et al.*, 2013 ▸). As elemental com­position, the nominal com­position was used in obtaining these PDFs. For *in situ* samples, a *Q*_min_ of 1 Å^−1^ and a *Q*_max_ of 14 Å^−1^ were used for the Fourier transform.

All refinements of PXRD data were performed using *TOPAS-Academic* (Version 7; Coelho, 2018 ▸). Analysis of PDF data was carried out using a modified version of the *DebyePDFGenerator* in *DiffPy-CMI* (Juhás *et al.*, 2015 ▸) to circumvent the Warren–Krutter–Morningstar approximation (Warren *et al.*, 1936 ▸) and to include the instrumental dampening of the PDF caused by Lorentzian broadening of the peaks in reciprocal space as suggested by Beyer *et al.* (2022 ▸). See Section S1 in the supporting information con­cerning modification.

As input structures for the three polymorphs of HfO_2_, *i.e.m*-HfO_2_, *t*-HfO_2_ and *c*-HfO_2_, the following entries in the Inorganic Crystal Structure Database (ICSD) were used; ICSD-142790 (*m*-HfO_2_) (Pathak *et al.*, 2020 ▸), ICSD-7146 (*t*-HfO_2_) (McCormack *et al.*, 2018 ▸) and ICSD-53033 (*c*-HfO_2_) (Jaffe *et al.*, 2005 ▸).

PXRD analysis of *ex situ* samples included either a single *c*-HfO_2_ phase or two phases of *m*-HfO_2_ and *c*-HfO_2_. The background was modelled using a measurement of an empty capillary, and to account for sample diffuse scattering, a multi-order Chebyshev polynomial and several broad Gaussian functions were used as well. Instrumental contributions to the peak profile were subtracted with an Si NIST SRM 640d measurement using the Thompson–Cox–Hastings pseudo-Voigt profile description. Size contributions to the peak profile were handled using whole powder pattern modelling (WPPM) assuming a lognormal size distribution of spherical particles using the built-in macro *WPPM_Sphere_LogNormDist* for samples annealed at 600 and 900 °C. The mean size and distribution width were extracted according to Scardi (2020 ▸) by 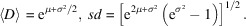


For samples annealed at 1200 °C, simultaneous refinement of a distribution width and size resulted in non-meaningful values and a simpler WPPM model assuming monodisperse spherical particles was used for these samples using the custom macro:

macro WPPM_Sphere_Diameter(SDc, SDv) {

#m_argu SDc

If_Prm_Eqn_Rpt(SDc, SDv, min. 1 max = Min(2 Val +. 3, 10000);)

WPPM_ft_conv = 1 − 1.5*WPPM_L/CeV(SDc, SDv) +

0.5*(WPPM_L/CeV(SDc, SDv))^3;

WPPM_break_on_small = 1e-7;

WPPM_L_max = 2*CeV(SDc, SDv);

WPPM_th2_range = 55;}

which is based on Equation (8) from Scardi & Leoni (2001 ▸).

Strain contributions were treated using the built-in macro *e0_from_Strain*. Other refined parameters include unit-cell parameters and ADPs. The occupancies of the ions in the *c*-HfO_2_ phase were adjusted according to Hf_1–*x*_Y_*x*_O_2–*x*/2_, with *x* being the Y^3+^ concentration as determined from ICP–OES (Table 1 ▸). Y^3+^ ions were assumed not to enter the *m*-HfO_2_ phase, and the Hf^4+^ and O^2−^ sites in this phase were assumed to be fully occupied. Atomic positions were fixed according to the input structures.

PXRD analysis of data from *in situ* experiments included either a single *c*-HfO_2_ phase or two phases, *m*-HfO_2_ and *c*-HfO_2_. In all experiments, an amorphous phase precipitates from the precursor solution as the heating commences, and this amorphous phase subsequently crystallizes into the *c*-HfO_2_ phase. To model the crystallization from the amorphous phase, the background is modelled using both a measurement of a capillary loaded with pure solvent heated to the same reaction tem­per­a­ture and one of the initial frames of each experiment, where only the amorphous precipitate is present. In addition to these, a fourth-order Chebyshev polynomial is included in the background description. To ensure a robust sequential refinement of the *in situ* data, apart from the background modelling, only the scale factors and coherent domain sizes (using the *CS_L* macro) of the crystalline phases were refined. Unit-cell parameters and ADPs were refined for the last frame in the experiment and fixed during the sequential refinement.

PDF analysis of the data from *in situ* experiments tracks the positions of select peaks, rather than a fully fledged model, to track the changes in the local structure. To extract the coherence length, *G*(*r*) in the range 5–50 Å was used, and a model using the *c*-HfO_2_ structure was employed. The refined parameters were scale, spherical dampening and the lattice parameter.

## 3. Results and discussion

**Figure 1:**
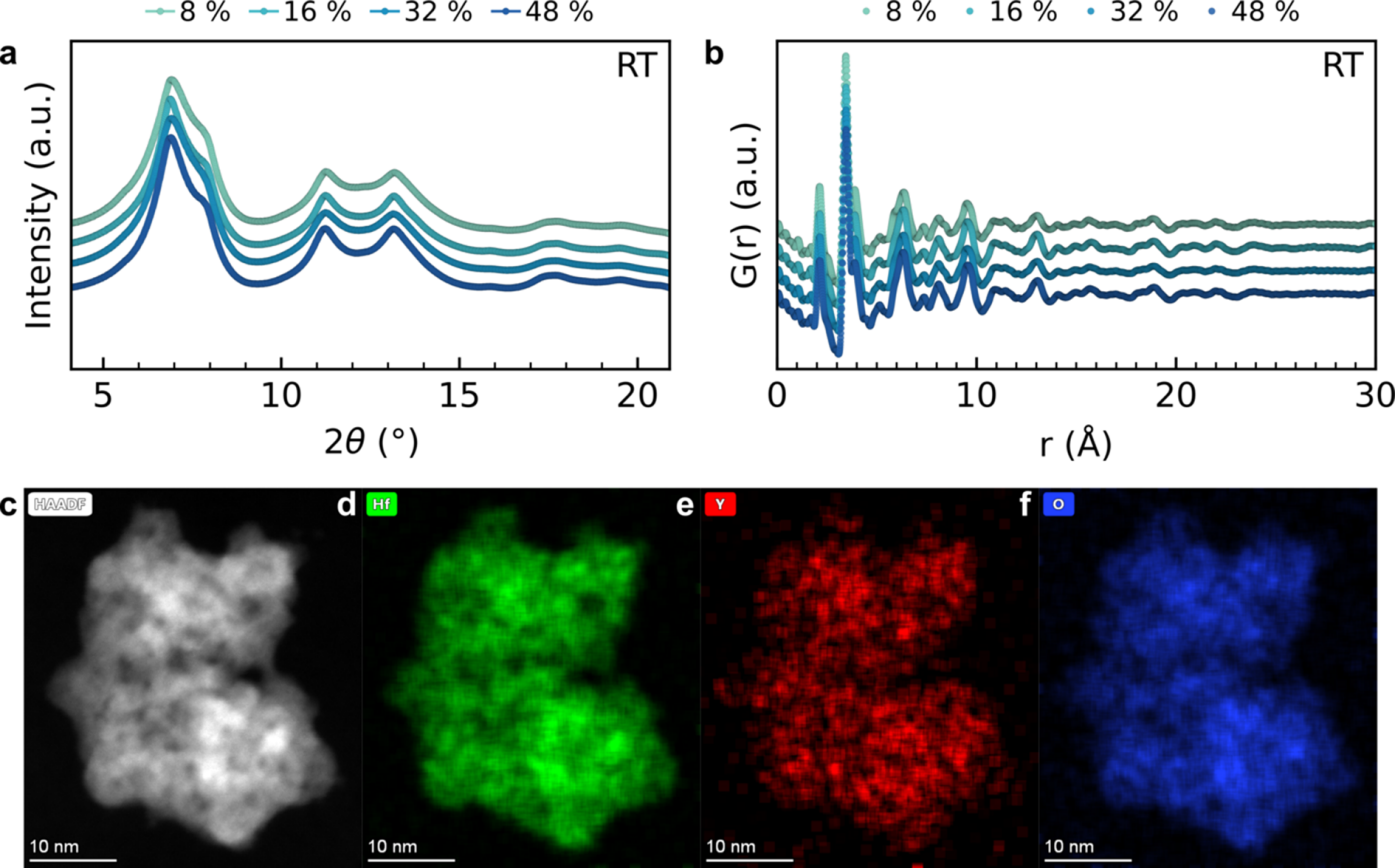
(*a*) PXRD patterns and (*b*) PDFs of the as-prepared YSH samples, and (*c*)–(*f*) STEM–EDX images of the as-prepared YSH48 sample. (*c*) HAADF image and corresponding EDX maps of (*d*) Hf, (*e*) Y and (*f*) O.

Powders of Hf_1–*x*_Y_*x*_O_2–*x*/2_ were synthesized with a continuous flow solvothermal synthesis reactor with nominal com­positions in the range *x* = 0.08–0.48, designated YSH*X* with *X* being the nominal atomic percentage of Y^3+^. The structural coherence of the as-prepared powders is very limited, as evidenced by the broad peaks in the PXRD patterns [Fig. 1 ▸(*a*)]. The peaks of the corresponding PDFs diminish around 15–20 Å, indicating that the structural coherence within the powders is not larger than the equivalent of ∼3–4 unit cells [Fig. 1 ▸(*b*)]. Not surprisingly, modelling the PXRD data in a Rietveld approach is unsuccessful, because the synthesized powders are borderline crystalline and may more appropriately be described as amorphous. Structurally, how­ever, the scattering patterns of the as-prepared powders share more resemblance with the *c*/*t*-HfO_2_ polymorph rather than the *m*-HfO_2_ polymorph, although neither phase adequately models the data (Fig. S8). Structural similarity between amorphous ZrO_2_ and its high-tem­per­a­ture polymorphs has been reported previously (Keramidas & White, 1974 ▸; Li Vage *et al.*, 1968 ▸; Zhang *et al.*, 2007 ▸).

The amorphous nature of the as-prepared powders is supported by STEM images of the samples, as the samples predominantly consist of larger agglomerates with no distinct discernible isolated particles [Fig. 1 ▸(*c*) for YSH48 and Figs. S9–S12 for the others]. As such, the vanishing peaks of the PDFs thus relate to the limited coherence of distinct local correlations within the borderline amorphous powders rather than size dampening from discrete nanoparticles.

For all samples, the experimental com­position deviates from the nominal com­position, revealing substanti­ally less Y^3+^ in the precipitated powders than anti­cipated (Table 1 ▸). This is most likely a result of incom­plete hydrolysis of the Y^3+^ ions during the continuous flow synthesis. Irrespective of this, the STEM–EDX maps show a homogenous distribution of Hf, Y and O within these agglomerates, confirming the formation of atomic-scale mixed Hf_1–*x*_Y_*x*_O_2–*x*/2_ powders that can serve as precursors for YSH nanoparticles in high-tem­per­a­ture annealing [Figs. 1 ▸(*d*)–(*f*) and S9–S12]. For YSH8, the ICP–OES com­positional analysis reveals a small impurity of Zr of ∼4 at% (Table S2) that is believed to originate from the HfCl_4_ precursor. The other samples contain less than 0.5 at% Zr. In the STEM–EDX map, Zr also seems to be homogenously distributed within the particle aggregates (Fig. S9). Since Hf and Zr are chemically similar, any impurity of Zr is expected to substitute randomly for Hf within the crystals.

**Figure 2:**
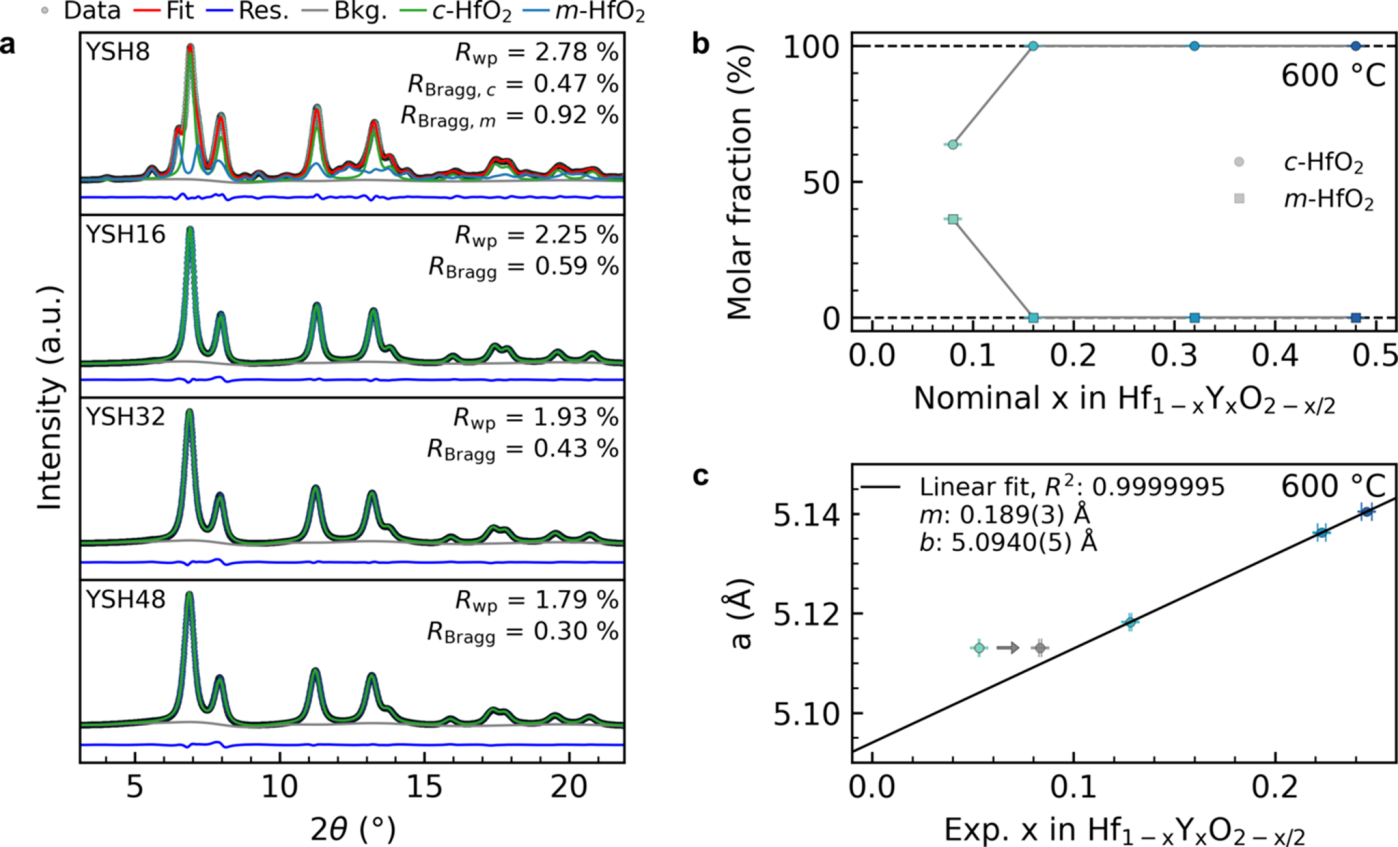
(*a*) Refined PXRD patterns of YSH samples annealed for 3 h at 600 °C. (*b*) Refined molar fractions against nominal com­position. (*c*) Refined unit-cell parameters of the *c*-HfO_2_ phase *versus* experimental Y^3+^ content, *x*, as determined from ICP–OES. The black line corresponds to the fit obtained from orthogonal distance linear regression to unit-cell parameters of phase-pure samples. The grey point corresponds to the unit cell of YSH8 moved horizontally on the com­position axis by dividing ICP–OES com­position with refined molar fraction of the *c*-HfO_2_ phase.

Serving as a precursor to the formation of nanocrystalline YSH by high-tem­per­a­ture annealing, the as-prepared amorphous powders with homogenous mixing of Hf^4+^ and Y^3+^ were annealed for 3 h at 600 °C. As evidenced by the PXRD patterns, this causes a crystallization of the amorphous pow­ders and formation of larger crystalline domains [Fig. 2 ▸(*a*)]. The decreased peak broadening allows for differentiation between cubic and tetra­gonal HfO_2_. As no peak splitting or asymmetry is observed, formation of *c*-HfO_2_ is concluded. For YSH8, a two-phase product of *c*-HfO_2_ and *m*-HfO_2_ is obtained with 36.3 (3)% *m*-HfO_2_, suggesting that a Y^3+^ content of 5.31 (3) at% is insufficient to fully stabilize the high-tem­per­a­ture phase of HfO_2_. In contrast, the PXRD patterns of the other samples can be modelled with a single *c*-HfO_2_ phase, demonstrating that the critical doping limit required for the com­plete stabilization of these nanosized samples lies somewhere between 5.31 (3) and 12.81 (5) at%. This is much lower than the limit suggested by Li *et al.* (2018 ▸) for microcrystalline YSH of 17 at%, but in agreement with other reports on nanocrystalline YSH that demonstrate com­plete stabilization with 10 at% (Zhou *et al.*, 2012 ▸; Qiu *et al.*, 2021 ▸).

The refined unit-cell parameters of *c*-HfO_2_ corroborate the homogeneous distribution of Hf^4+^ and Y^3+^ in the as-prepared amorphous powders. The unit-cell parameters demonstrate Vegard-like behaviour, *i.e.* a linear increase against the ex­peri­mentally determined Y^3+^ content [Fig. 2 ▸(*c*)], in accordance with the effective ionic radii of Hf^4+^ and Y^3+^ of 0.83 and 1.019 Å (Shannon, 1976 ▸).

The unit-cell parameters of the phase-pure samples fall on a perfectly straight line. Orthogonal distance linear regression to the data points suggests a hypothetical unit-cell parameter of undoped *c*-HfO_2_ of 5.0940 (5) Å, based on the inter­cept, and a slope of 0.189 (3) Å. Since undoped *c*-HfO_2_ is rarely stabilized at room tem­per­a­ture, only a few accounts of the unit-cell parameters without dopants are reported with values in the range 5.12–5.129 Å for non-stoichiometric HfO_2–δ_ (Lu *et al.*, 2008 ▸; Manory *et al.*, 2002 ▸). These values are slightly higher than the value extracted from Vegard-type fitting here. The non-stoichiometric nature of the undoped cubic HfO_2_ could explain the discrepancy with Vegard-type fitting, as oxygen vacancies have been linked to enlarged lattice parameters (Marrocchelli *et al.*, 2012 ▸). In the literature on Y^3+^ doping of HfO_2_, it is customary to perform the Vegard analysis of the refined unit-cell parameters against the molar percentage of Y_2_O_3_ in the sample, *i.e.* (HfO_2_)_1–*y*_(Y_2_O_3_)_*y*/2_, rather than Hf_1–*x*_Y_*x*_O_2–*x*/2_. Thus, for com­parative purposes, the same Vegard analysis is performed when plotted against mol% Y_2_O_3_, yielding an inter­cept of 5.0969 Å and a slope of 3.12 (5) × 10^−3^ Å mol%^−1^ (Fig. S13). These values are in reasonable agreement with the literature values of 5.095–5.106 Å and 2.5 × 10^−3^–2.9 × 10^−3^ Å mol%^−1^, respectively, although the slope obtained here is slightly higher (Kim, 1989 ▸; Sévin *et al.*, 2020 ▸; Weyl & Janke, 1996 ▸).

Normalizing the com­position of YSH8 by the refined molar fraction of the *c*-HfO_2_ phase almost moves its unit cell onto the straight line [grey point, Fig. 2 ▸(*c*)], suggesting that phase-segregated domains of *m*-HfO_2_ are predominantly pure HfO_2_ and that the Y^3+^ remains in the *c*-HfO_2_ structure. If the Y^3+^ ions entered the *m*-HfO_2_ structure, this normalization would have moved the point to the right of the Vegard line. The remaining discrepancy, even after normalizing with the refined molar fraction, may be linked to the small amount of Zr^4+^ detected in this sample [Fig. S13(*b*)], given its slightly larger ionic radius of 0.84 Å com­pared with 0.83 Å for Hf^4+^ (Shannon, 1976 ▸). Slight uncertainties in the estimations of both molar fraction and unit cell are equally possible explanations for this discrepancy, since all peaks from the *c*-HfO_2_ phase overlap with some from the *m*-HfO_2_ phase.

Annealing the samples at higher tem­per­a­tures causes continued growth of the nanoparticles and induces progressive phase segregation (Figs. S14–S19). As mentioned, no distinct particles are found in the as-prepared samples, but after annealing at 600 °C, distinct regions resembling small crystallites are found. These regions appear larger and more coherent after 900 °C and fully crystalline particles are found after annealing at 1200 °C. At 1200 °C, even the most doped samples phase segregate, although the refined molar fractions of *m*-HfO_2_ are small, with values of 6.23 (9) and 2.29 (10)% for YSH32 and YSH48, respectively. A Y-segregation is identified using STEM–EDX of sample YSH16 annealed at 1200 °C supporting a phase segregation into Y-rich and Y-poor regions (Fig. S20), likely corresponding to *c*-HfO_2_ and *m*-HfO_2_, respectively. The annealing series re-emphasizes that the stabilization of the *c*-HfO_2_ crystal structure is not exclusively driven by the creation of oxygen vacancies resulting from Y^3+^ incorporation into the lattice. This is evidenced by the fact that the *c*-HfO_2_ phase stabilizes at doping levels lower than the critical limit reported for microcrystalline samples.

Zhou *et al.* (2012 ▸) observed similar stabilization in 10–12 nm-sized nanocrystalline samples at a doping level of 10 at%, whereas Qiu *et al.* (2021 ▸) found com­plete stabilization in 52 nm nanoparticles at 10 at%. Here, coherent domain sizes are extracted using WPPM modelling of the PXRD patterns assuming lognormal distribution of spherical particles. The refined mean sizes and distribution widths can be found in Table S3 and are visualized in Fig. S21. In com­parison with the results of Qiu *et al.* (2021 ▸), the YSH16 sample with an actual com­position of 12.81 (5) at% is com­pletely stabilized in 5.4 (1) nm nanoparticles (average size from WPPM) when annealed at 600 °C, but phase segregation occurs in 9.8 (2) nm nanoparticles when annealed at 900 °C, *i.e.* in much smaller particles, even when considering the overall lognormal distribution.

Both Zhou *et al.* (2012 ▸) and Qiu *et al.* (2021 ▸) argued that lower surface free energy of the high-tem­per­a­ture phases com­pared with the thermodynamically stable *m*-HfO_2_ is responsible for its stabilization at the nanoscale. The idea of a ‘size effect’ theory was initially put forth by Garvie (1965 ▸) for undoped ZrO_2_, and it has later been predicted for HfO_2_ as well (Sharma *et al.*, 2018 ▸). Others suggest that instead, intrinsic oxygen-ion vacancies are responsible for the stabilization in undoped ZrO_2_ and that the elimination of these, rather than the domain growth itself at elevated tem­per­a­tures, initiates phase segregation (Osendi *et al.*, 1985 ▸; Liu *et al.*, 1995 ▸). Again, this has been noted for undoped HfO_2_ as well (Lu *et al.*, 2008 ▸; Kumar *et al.*, 2017 ▸). The apparent effect of the preparation method, *i.e.* this study com­pared with previous studies, on the size and doping concentration relationship would further support this conjecture. As such, under-coordinated metal ions in the as-prepared amorphous powders are a potential source of additional oxygen-vacancy defects in the annealed samples pre­sent­ed here.

### 3.1. Doping-induced local disorder

The long-range order, as reflected in the PXRD patterns of all YSH samples, is well-modelled in Rietveld refinements with *c*-HfO_2_ only or in combination with *m*-HfO_2_, as demonstrated in Figs. 2 ▸ and S14. The refinements, how­ever, also result in unusually large ADPs for both metal and oxygen sites (Fig. S22). Such high ADP values suggest that the long-range structural signal of the PXRD patterns is dampened, not only by the Debye–Waller factor from atomic thermal motion or factors such as absorption from the sample, but additionally by structural disorder. This is further corroborated by diffuse scattering contributing to the background of the PXRD patterns, apart from the scattering of the sample container [Fig. S23(*a*)], indicating correlated disorder within the YSH samples.

**Figure 3:**
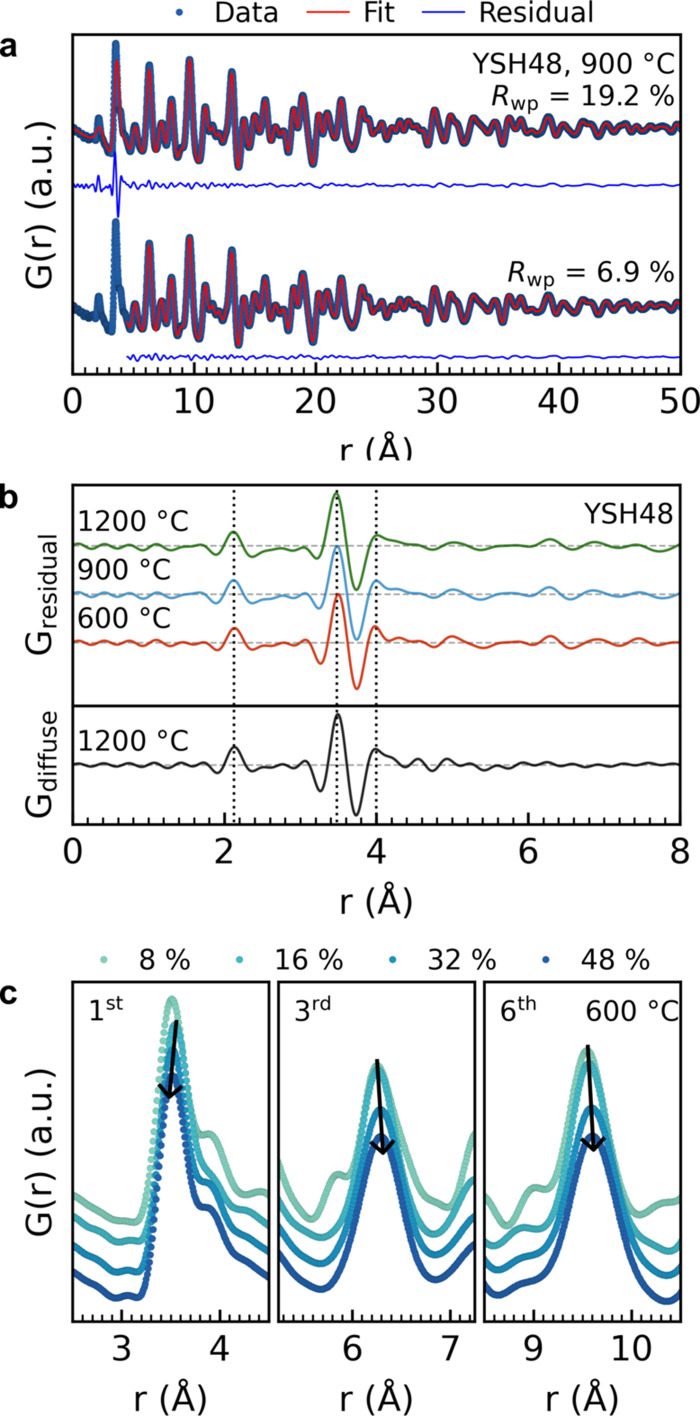
(*a*) PDF of the YSH48 sample annealed at 900 °C modelled in the ranges 0–50 (top) and 4.5–50 Å (bottom) with the *c*-HfO_2_ crystal structure. (*b*) Comparison of (top) the PDF residual curves from 0–50 Å fits of YSH48 samples annealed at 600, 900 and 1200 °C, and (bottom) the PDF obtained by Fourier transformation of the diffuse contribution from Rietveld refinement of the YSH48 sample annealed at 1200 °C (see Fig. S23). (*c*) Zoom-ins of the first, third and sixth *M*—*M* (*M* = Hf^4+^, Y^3+^) peaks in the PDFs of samples annealed at 600 °C. Black arrows indicate the shift in peak position as the Y^3+^ concentration is increased.

Both effects are reflected in the corresponding PDFs and manifest themselves in high values of refined ADPs (Fig. S24) and structurally in the very-short-range region of the PDFs below 4.5 Å (Fig. 3 ▸). The PDF of YSH48 annealed at 900 °C exemplifies this, as the *c*-HfO_2_ phase provides an excellent description of all correlations above 4.5 Å, but the model is unable to fully describe the nearest-neighbour (NN) *M*—O and *M*—*M* correlations at 2.15 and 3.55 Å, respectively. The absence of any structural signal in the residual curve above the short-range region rules out the possibility of significant undetected crystalline impurity phases being responsible for the signal. Consequently, modelling only the PDF above 4.5 Å leads to an *R*_wp_ of 6.9%, com­pared with 19.2% for the full-range fit. The ADPs from the PDF refinements of the metal and oxygen site are 0.0277 (6) and 0.056 (6) Å^2^, respectively, in line with the results from the reciprocal-space refinements.

The residual curve in the full-range fit shows a predominant ‘positive–negative–positive’ feature around the NN *M*—*M* peak [Fig. 3 ▸(*a*)], which is an indication of displacive disorder (Støckler *et al.*, 2024 ▸). This feature is also present at the other annealing tem­per­a­tures [Fig. 3 ▸(*b*), top] and in the other phase-pure samples (Fig. S25). In fact, by separating the diffuse scattering from the other non-Bragg scattering contributions to the PXRD patterns and Fourier transforming this yields a PDF reproducing the ‘positive–negative–positive’ feature of the residual curves (see Fig. S23). This is exemplified for YSH48 annealed at 1200 °C [Fig. 3 ▸(*b*), bottom]. Combined, this suggests static displacive disorder within the metal sublattice that decreases the distance to some of the neighbouring metal ions while increasing it to others.

Comparing the first, third and sixth nearest *M*—*M* correlations in the PDFs of the phase-pure samples annealed at 600 °C (YSH16–48) strongly suggests that the displacive disorder is correlated with the Y^3+^ doping [Fig. 3 ▸(*c*)]. Note that other *M*—O and O—O correlations will also contribute to the third and sixth *M*—*M* correlations. As pre­sent­ed in Fig. 2 ▸(*c*), increasing the Y^3+^ dopant concentration expands the *c*-HfO_2_ unit cell consistent with Vegard’s law, and the same expansion is reflected in the slight rightward shift of the third and sixth *M*—*M* correlation peaks in the PDFs as the dopant concentration increases [Fig. 3 ▸(*c*)]. Counterintuitively, how­ever, the first *M*—*M* peak shifts in the opposite direction, indicating a greater degree of disorder as the dopant concentration is increased.

In YSZ, it has been shown that, by virtue of its larger ionic radius, Y^3+^ more easily accommodates the eight­fold coordination of the cubic fluorite structure, leaving the generated oxygen-ion vacancies for the smaller Zr^4+^ ions that consequently adopt their preferred CN of seven of the thermodynamically stable monoclinic structure (Li *et al.*, 1994*b* ▸; Khan *et al.*, 1998 ▸; Ho, 1982 ▸; Fèvre *et al.*, 2005 ▸). Studies of the diffuse scattering from single crystals of YSZ suggest local relaxation of the ions neighbouring the vacancies, with NN oxygen ions moving towards the vacancy along the 〈100〉 direction and NN Zr^4+^ ions moving away from the vacancy along 〈111〉 (Schmidt *et al.*, 2023 ▸; Fèvre *et al.*, 2005 ▸; Kaiser-Bischoff *et al.*, 2005 ▸; Frey *et al.*, 2005 ▸). Electrostatically, such relaxation is expected, as the negatively charged oxygen ions are attracted to the net positive oxygen-ion vacancies, whereas the positively charged Zr^4+^ ions are repelled. In the present case, a static displacement of the Hf^4+^ ions along 〈111〉 will inevitably introduce shorter and longer bonds to all neighbouring metal ions, in agreement with the ‘positive–negative–positive’ feature, thus a relaxation scheme similar to YSZ is likely to be responsible for the signal in these YSH samples.

**Figure 4:**
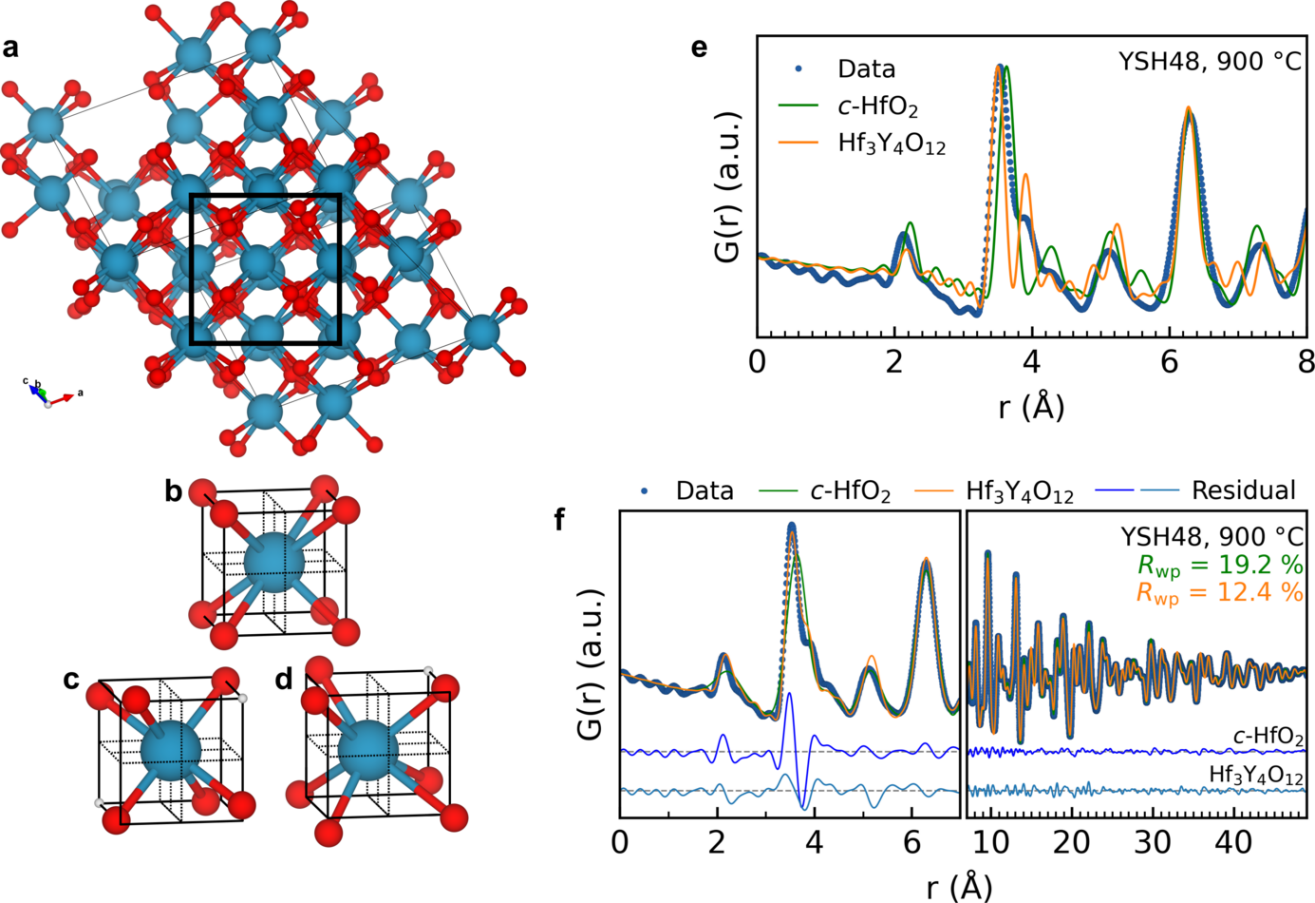
(*a*) The crystal structure of Hf_3_Y_4_O_12_ overlaid with a unit cell (black) corresponding to the *c*-HfO_2_ unit cell. (*b*)–(*d*) Comparison of the cation coordination in (*b*) the ordered fluorite structure with (*c*) sixfold (3*a*) and (*d*) sevenfold (18*f*) coordinated sites in Hf_3_Y_4_O_12_. (*e*) The PDF of YSH48 annealed at 900 °C overlaid with calculated PDFs of *c*-HfO_2_ (green) and Hf_3_Y_4_O_12_ (orange), with adjusted unit-cell parameters to com­ply with the experimental PDF, but assuming atomic positions from Zr_3_Y_4_O_12_ reported by Scott (1977 ▸). (*f*) Comparison of the full-range modelling (0–50 Å) of the *c*-HfO_2_ and Hf_3_Y_4_O_12_ phases.

Fèvre *et al.* (2005 ▸) showed that the diffuse scattering from single crystals of highly doped YSZ (∼18 to ∼43 at% Y^3+^) gradually condenses into broad Bragg-like features in 3D reciprocal space that can be indexed by a δ-phase with a com­position of Zr_3_Y_4_O_12_ (space group *R*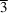) of the Y^3+^-rich part of the ZrO_2_–Y_2_O_3_ system. The crystal structure of Zr_3_Y_4_O_12_ was initially reported by Scott (1977 ▸), but is also known from the ZrO_2_–Sc_2_O_3_, HfO_2_–Sc_3_O_3_ and other systems (Thornber *et al.*, 1968 ▸; Rossell, 1976 ▸). Structurally, the δ-phase resembles the cubic fluorite structure, and it may best be described as a vacancy defect and disordered fluorite-type structure, as visualized in Fig. 4 ▸(*a*). Here, its crystal structure is visualized down the [2
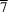
6] direction and overlaid with a black square emphasizing its resemblance with the cubic fluorite unit cell. In the δ-phase, the two types of cations are randomly distributed, but the vacancies order to give two distinct types of cation coordination sites: sixfold coordinated 3*a* sites [Fig. 4 ▸(*c*)] and sevenfold coordinated 18*f* sites [Fig. 4 ▸(*d*)]. These sites are distorted relative to the eightfold-coordinated ideal square-prismatic coordination of the fluorite structure [Fig. 4 ▸(*b*)]. This is highlighted by the vertices of the cube, marking the oxygen positions in the ideal fluorite square prism, and the dashed guidelines indicating the central cation position in Figs. 4 ▸(*b*)–(*d*). The vacancies around both sites are illustrated with small white spheres at the vertices of the cube.

Evidently, both cations and anions relax around the vacancies, seemingly following the same rules as derived from single-crystal diffuse scattering and electrostatic considerations: the NN oxygen ions displace directly towards the vacancy and the NN cations at the 18*f* site displace away from the vacancy along what would be the 〈100〉 and 〈111〉 directions of the fluorite lattice, respectively. Since the 3*a* sites have vacancies at opposing sides of the cation, the 3*a* cation does not displace.

In Fig. 4 ▸(*e*), calculated PDFs of the *c*-HfO_2_ and *δ*-phase (Hf_3_Y_4_O_12_) are com­pared to the low *r* region of the experimental PDF of the YSH48 sample annealed at 900 °C. The first *M*—*M* peak of the *c*-HfO_2_ phase splits into two distinct distances for Hf_3_Y_4_O_12_, which matches well with the features of the experimental PDF that give rise to the ‘positive–negative–positive’ features in the residual curves in Fig. 3 ▸(*b*). This suggests that the similar local relaxation around vacancies observed in single-crystal diffuse scattering for YSZ may also be at play in the nanoparticles of YSH.

Full-range modelling of the PDF with either *c*-HfO_2_ or Hf_3_Y_4_O_12_ provides similar descriptions of the PDF above 5.5 Å [Fig. 4 ▸(*f*)], and if the modelling is performed in the range 4.5–50 Å, the calculated PDFs become visually inseparable (Fig. S26). This reiterates that the δ-phase is simply a distorted and disordered fluorite-type structure. Yet, the Hf_3_Y_4_O_12_ structure provides a better description of the first *M*—O and *M*—*M* correlations [Fig. 4 ▸(*f*)]. With the Hf_3_Y_4_O_12_ structure, the refined ADPs decrease to 0.01983 (7) and 0.020 (7) Å^2^ for metal and oxygen sites (Table S4), respectively, essentially showing that the structural disorder is decoupled from the atomic thermal motion with this model. Although it leads to an improved description of the PDF, the Hf_3_Y_4_O_12_ structure does not com­ply with the PXRD patterns of the YSH samples, as the lower symmetry of the trigonal structure dictates additional peaks in the calculated patterns which are not present in the experimental data (Fig. S27). This emphasizes that the actual relaxation around the vacancies is more com­plex and disordered than what the average Hf_3_Y_4_O_12_ crystalline model can represent, because the relaxation around the vacancies is uncorrelated and thus does not possess long-range order. However, the local coordination environment of the δ-phase resembles the local disorder in the YSH nanoparticles.

The lower thermal con­duc­tivity of YSH com­pared to undoped *m*-HfO_2_ is commonly ascribed to phonon scattering by oxygen vacancies and mass disorder from Y^3+^ ion substitution on Hf^4+^ sites (Ramana *et al.*, 2012 ▸; Winter & Clarke, 2006 ▸; Klemens & Gell, 1998 ▸). However, the identification of vacancy-associated displacive disorder in these samples suggests that local structural distortions surrounding the vacancies may also inhibit phonon propagation and thus contribute to the com­paratively low thermal con­duc­tivity.

### 3.2. Crystallization mechanism

To shed light on the crystallization process of the amorphous YSH powders, the synthesis of Hf_1–*x*_Y_*x*_O_2–*x*/2_ nanoparticles was investigated with *in situ* PXRD during solvothermal synthesis. Here, precursor solutions similar to those used in the continuous flow syntheses of the *ex situ* powders are loaded into a reactor capillary and heated rapidly to the desired synthesis tem­per­a­ture, while simultaneously collecting X-ray scattering data suitable for Rietveld and PDF analysis. A total of eight *in situ* experiments, covering the com­position series of Hf_1–*x*_Y_*x*_O_2–*x*/2_ for *x* = 0.00–0.32 and a tem­per­a­ture series in the range 250–400 °C (*x* = 0.24), have been performed. An overview of the experiments in terms of contour plots and selected refined frames can be found in Figs. S28–S32.

**Figure 5:**
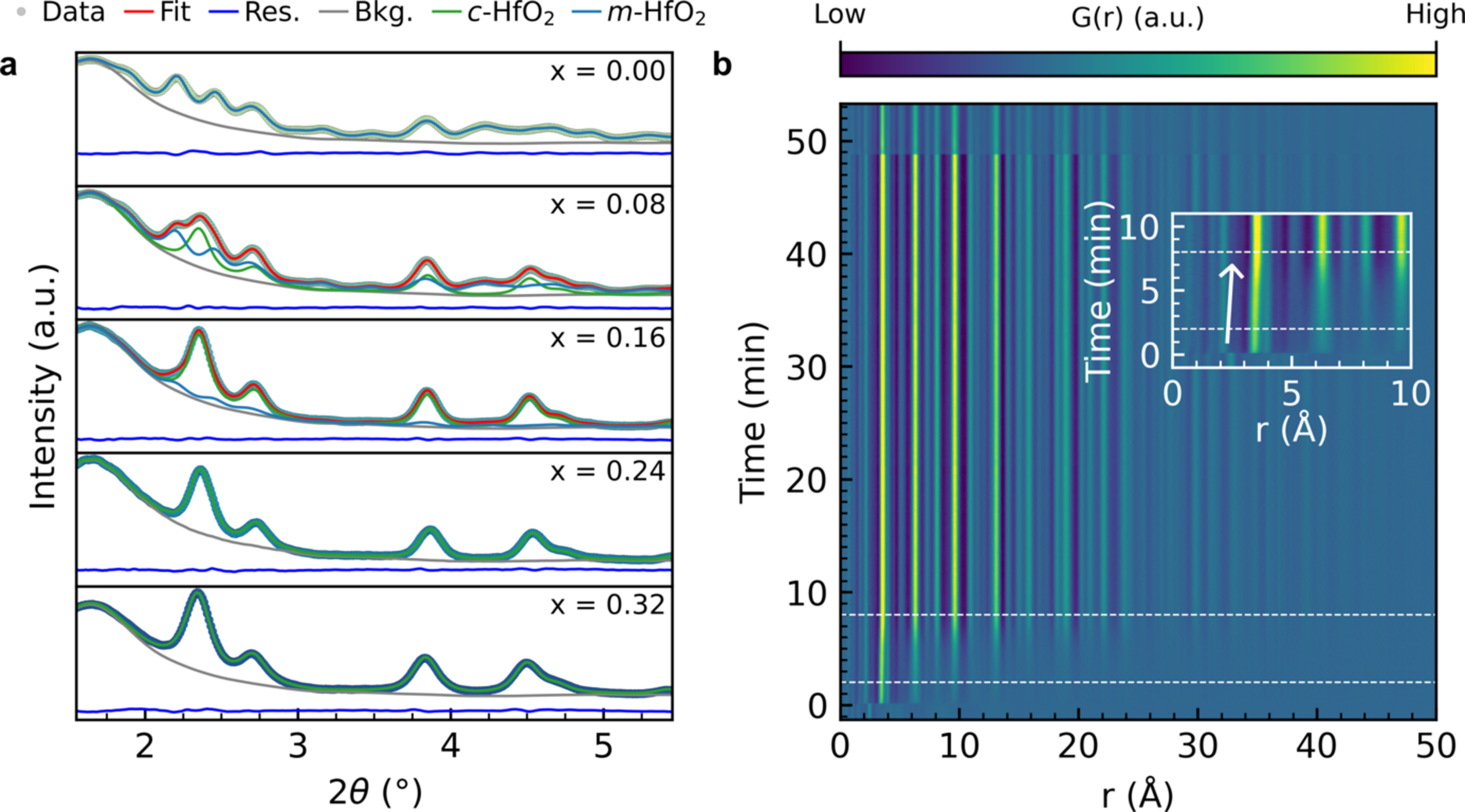
(*a*) Refined PXRD patterns from *in situ* syntheses for all com­positions after 20 min of synthesis. (*b*) 2D contour plot of PDFs during the *in situ* experiment of *x* = 0.24. The inset shows the first 10 min of the experiment.

The refined PXRD patterns after 20 min of synthesis demonstrate the simultaneous incorporation of Hf^4+^ and Y^3+^ in the HfO_2_ lattice for all Y^3+^-doped samples [Fig. 5 ▸(*a*)], as the *c*-HfO_2_ phase forms in all experiments, except the control experiment of undoped HfO_2_, where phase-pure *m*-HfO_2_ is obtained. At nominal com­positions of *x* = 0.08 and 0.16, the amount of Y^3+^ is insufficient to com­pletely stabilize the *c*-HfO_2_ phase, and during the experiments, increasing amounts of the *m*-HfO_2_ phase segregate (Fig. S33). Like for the *ex situ* samples, not all the Y^3+^ ions from the precursor solutions are hydrolysed and incorporated into the *c*-HfO_2_ structure, giving rise to non-linear dependence of the room-tem­per­a­ture unit-cell parameters of the *c*-HfO_2_ phase against nominal com­position (Fig. S34).

Based on contour plots of the PDFs of the *in situ* experiments, the synthesis of the YSH nanoparticles can be divided into three stages, regardless of the phase purity of the products, with an example shown for *x* = 0.24 in Fig. 5 ▸(*b*) and for other samples in Figs. S35–S36. Before heating, only the precursor solution with limited structural extent is present, but as soon as heating commences (*t* = 0), an immediate change in the local coordination occurs, with the structural signal extending substanti­ally further than that of the precursor solution. The structural extent, how­ever, is still limited to less than ∼20 Å, and the similarity with the PDF of the as-prepared *ex situ* samples is striking (Fig. S37). Photos monitoring the reactor capillary during the *in situ* experiment reveal that within seconds after heating, the initially translucent precursor solution inside the capillary turns white (Fig. S38). Together these observations suggest that the changed coordination in the PDF reflects the precipitation of an amorphous solid from the solution.

**Figure 6:**
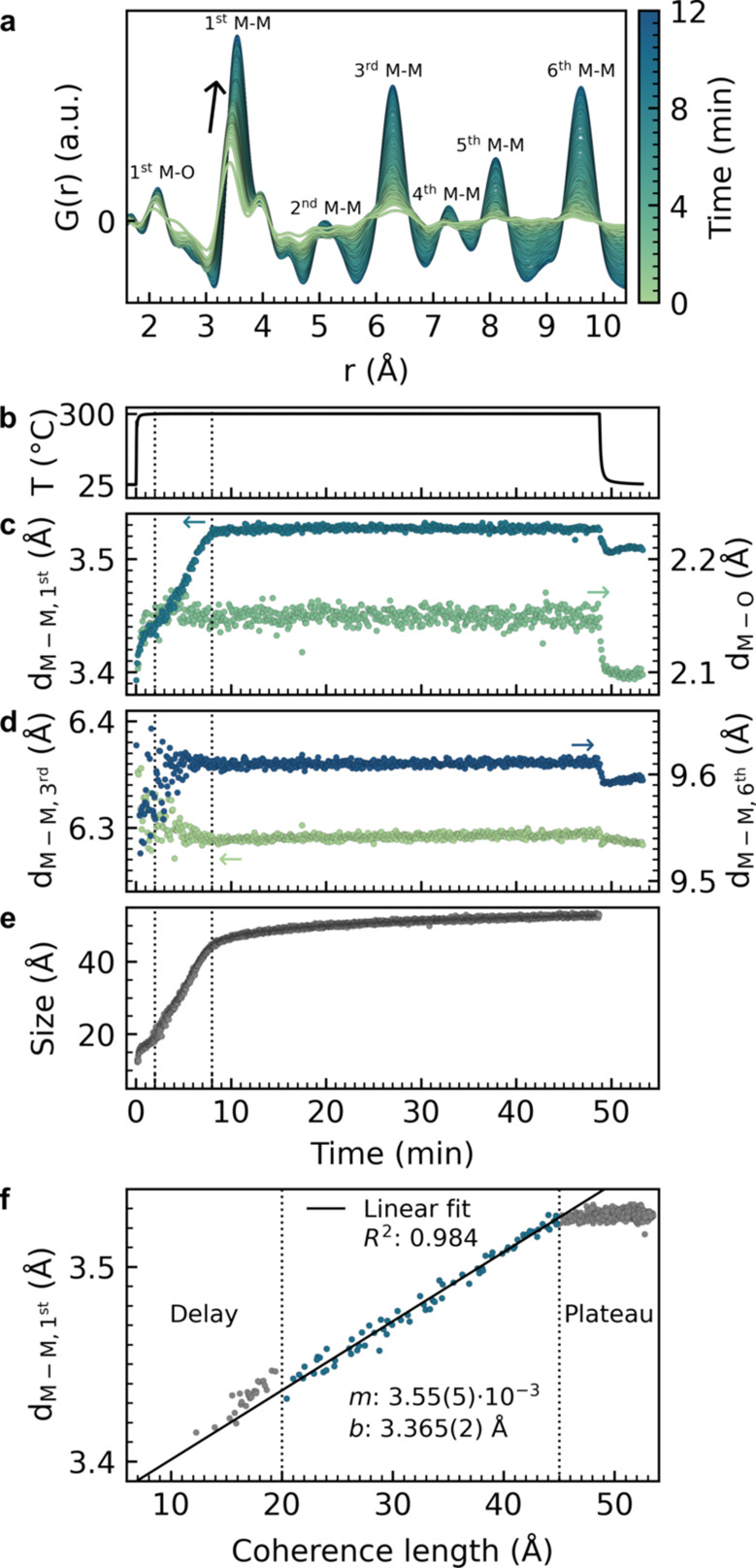
Local changes in the short-range region of the PDF during the *in situ* solvothermal synthesis experiment (*x* = 0.24). (*a*) Low-*r* region of the raw PDFs. (*b*) Heating profile during the experiment, together with temporal evolution in peak position of (*c*) the first *M*—O and first *M*—*M* distances, (*d*) the third and sixth *M*—*M* distances, and (*e*) the refined coherent domain size, *i.e.* coherence length in the PDF. (*f*) The first *M*—*M* distance *versus* coherence length. The vertical dashed lines in (*b*)–(*f*) mark the end of an initial delay period before the first *M*—*M* distance expansion and the end of the gradual *M*—*M* expansion.

Entering the second stage of the synthesis, structural co­herence within the amorphous precipitate builds and a clear expansion of the first *M*—*M* correlations occurs [inset, Fig. 5 ▸(*b*)]. As the expansion concludes, the third stage of the synthesis begins, characterized by only modest changes in the PDFs. The local changes occurring during the second stage of the synthesis are explored in greater detail in Fig. 6 ▸. In Fig. 6 ▸(*a*), the low-*r* region of the PDFs is shown as a waterfall plot, with their colours indicating the progression of the synthesis. Here, the rightward shift of the first *M*—*M* distance and the simultaneous intensity increase of the longer *M*—*M* peaks is clearly visualized.

By tracking the position of peak maxima *via* single peak fitting of the first *M*—*M* peak, it appears that following an initial rapid increase, which coincides with the point of heating, this is followed by a corresponding increase in the first *M*—O distance, and a short delay period impedes the subsequent transformation [Figs. 6 ▸(*b*)–(*c*)]. However, as this delay concludes, the first *M*—*M* distance continuously expands [Fig. 6 ▸(*c*)] and is mirrored by a corresponding increase in the refined coherent domain size [Fig. 6 ▸(*e*)]. The increase in coherence length is also reflected in the other *M*—*M* distances that sharpen and increase in intensity during this period [Fig. 6 ▸(*a*)]. After approximately 8 min, the first *M*—*M* distance levels at a value of ∼3.53 Å, which is in line with the annealed *ex situ* YSH samples [Fig. 3 ▸(*c*)]. Domain growth, on the other hand, continues, albeit at a much-reduced rate. The relatively low *Q*_max_ limits the ability to resolve features in the PDF; how­ever, in this case, as relative changes of the same correlation peaks are reported, this is less of an issue.

Plotting the peak position of the first *M*—*M* distance against the refined coherent domain size shows that the change in the *M*—*M* distance correlates linearly with the structural coherence length observed in the PDF, until the *M*—*M* distance levels at a coherent domain size of ∼4.5 nm [Fig. 6 ▸(*e*)]. This suggests that the observed reorganization reflects gradual ordering of the atoms within the amorphous precipitate, *i.e.* crystallization, rather than incorporation of more Y^3+^ ions in the structure. Similar crystallization from an amorphous matrix has been reported for the solvothermal synthesis of YSZ nanoparticles with a 15 at% Y^3+^ dopant concentration, also revealed by *in situ* PDF analysis (Tyrsted *et al.*, 2014 ▸).

Increasing the synthesis tem­per­a­ture increases the rate at which long-range order builds within the precipitate (Fig. S39). At 400 °C, the ordering occurs within a minute, whereas lowering the tem­per­a­ture to 250 °C impedes the transformation and a plateau is reached after 30 min. With more thermal energy available at higher tem­per­a­tures, diffusion of the ions within the precipitate increases, thus facilitating enhanced local reorganization and ordering.

Inter­estingly, the degree of Y^3+^ doping also directly influences the crystallization kinetics by slowing the structural reorganization at increasing Y^3+^ concentration (Fig. S40). The time it takes for the first *M*—*M* distance to plateau increases substanti­ally with nominal Y^3+^ concentration. For the phase-segregated samples, the average first *M*—*M* distance does not stay constant after the second stage of the synthesis has been concluded, but rather contracts slightly over the remainder of the experiment [Figs. S40(*b*)–(*c*)]. Since this correlates with the emergence of the second *m*-HfO_2_ phase [*cf*. Figs. S33(*b*)–(*c*) and S40(*b*)–(*c*)], it simply reflects the fact that the shortest *M*—*M* distance in *m*-HfO_2_ is slightly shorter than for the *c*-HfO_2_ phase. As more *m*-HfO_2_ phase segregates, the average *M*—*M* distance gradually decreases.

A substantial amount of time is required for com­plete ordering to occur at the local scale, and this correlates well with the amorphous nature of the YSH samples from the continuous flow syntheses, as the short residence time within the reactor (∼39 s) is insufficient to rearrange the atoms of the amorphous precipitate and achieve long-range crystalline order. Instead, substanti­ally longer residence time or higher reaction tem­per­a­tures would be required to obtain crystalline YSH nanoparticles from this one-step continuous flow synthesis method. For the as-prepared *ex situ* YSH samples, the thermal energy required for crystallization is supplied by annealing at 600–1200 °C, but the mechanism of local ordering is expected to be similar.

## 4. Conclusion

Homogenous amorphous YSH powders with variable Y^3+^ contents were obtained *via* continuous flow solvothermal synthesis using supercritical ethanol as reaction medium. The hydrolysis of the Y^3+^ ions was insufficient and less-than-nominal amounts of Y^3+^ were incorporated into the powders. Subsequent high-tem­per­a­ture annealing of the obtained amorphous powders induced crystallization, and this demonstrates the stabilizing effect of Y^3+^ doping on the high-tem­per­a­ture *c*-HfO_2_ polymorph at room tem­per­a­ture. The refined unit-cell parameters conform to a Vegard-like relationship against experimentally determined Y^3+^ content, reflecting the homogeneity of the samples. Full stabilization of the *c*-HfO_2_ phase was achieved at a doping level of 13 at% Y^3+^, which is substanti­ally lower than the suggested critical doping level in bulk YSH of ∼17 at%. This is attributed to non-doping-induced oxygen-ion vacancies that, together with the doping-induced vacancies, co-operatively stabilize the *c*-HfO_2_ phase at lower Y^3+^ concentrations.

While the long-range order of the annealed powders conforms to the ordered fluorite structure of *c*-HfO_2_, as demonstrated by Rietveld refinements of the PXRD patterns, the high ADP values of both metal and oxygen sites and the presence of diffuse scattering suggest local disorder of the structure. A deeper investigation of the local structure of the particles using PDF analysis reveals that, while the average long-range order conforms to the fluorite structure, the structure is locally disordered, and that the degree of disorder correlates with the degree of doping. The local disorder can be reasoned by electrostatic relaxation around the generated oxygen-ion vacancies, as previously identified in single crystals of YSZ. A model based on the well-known Zr_3_Y_4_O_12_ structure, which incorporates such relaxation motifs, provides a description of the main residual features from the PDF analysis. It is hypothesized that this local relaxation of the ions surrounding the oxygen vacancies will also affect phonon scattering and thus, together with mass disorder and vacancy point defects, contribute to the low thermal con­duc­tivity of YSH com­pared to undoped HfO_2_.

Investigation of the solvothermal synthesis of YSH using *in situ* total scattering experiments explains the amorphous nature of the as-synthesized powders, as the crystallization of the initially amorphous precipitate into the locally disordered *c*-HfO_2_ phase occurs over the course of several minutes, whereas the reaction time of the *ex situ* powders was only ∼40 s. The structural reorganization of the local structure during the crystallization is tracked *via* single peak fitting of the PDFs, and inter­estingly the rate of crystallization depends not only on the synthesis tem­per­a­ture, but also on the degree of doping. Higher reaction tem­per­a­tures result in faster crystallization and larger degrees of doping result in slower crystallization.

## Supplementary Material

Supporting information. DOI: 10.1107/S2052252526003829/fc5086sup1.pdf

## Figures and Tables

**Table 1:** Experimental com­position as determined by ICP–OES

Sample name	Nominal com­position (at% Y)	ICP–OES com­position (at% Y)
YSH8	8	5.31 (3)
YSH16	16	12.81 (5)
YSH32	32	22.3 (2)
YSH48	48	24.5 (3)

## Data Availability

The data are available from the authors upon request.
