# Supplementary material for: Defect structure of yttria-stabilized hafnia nanoparticles
Source: IUCrJ. 2026 Jun 9;13(Pt 4):485–97. doi: 10.1107/S2052252526003829 (PMC13324614; doi:10.1107/S2052252526003829)
Supplement: Supplementary file 1 [file m-13-00485-sup1.pdf]

IUCrJ

Volume 13 (2026)

Supporting information for article:

Defect structure of yttria-stabilized hafnia nanoparticles

Magnus Nørgaard Kløve, Andreas Dueholm Bertelsen, Mads Ry Vogel Jørgensen and Bo Brummerstedt Iversen

Supporting information

Defect Structure of Yttria-Stabilized Hafnia Nanoparticles

Magnus Nørgaard Kløve,[a] Andreas Dueholm Bertelsen,[a] Mads Ry Vogel Jørgensen,[a,b] and Bo Brummerstedt Iversen*[a]

[a] Center for Sustainable Energy Materials, Department of Chemistry, Aarhus University, Langelandsgade 130, 8000 Aarhus C, Denmark

[b] MAX IV Laboratory, Lund University, Fotongatan 224 84 Lund, Sweden

*Correspondence e-mail: bo@chem.au.dk

S1. Modelling PDFs in DiffPy-CMI with modified version of DebyePDFGenerator

Refinements of the PDF for *ex situ* samples were carried out using the DiffPy-CMI modelling software (Juhás *et al.*, 2015*b*). To ensure accurate calculation of the PDFs by avoiding the Warren-Krutter-Morningstar approximation (Warren *et al.*, 1936), the calculated PDFs were generated using the Debye scattering equation (DSE) and subsequent Fourier transformation to direct space, by utilizing the *DebyePDFGenerator()* already implemented in DiffPy-CMI. The calculated PDFs were modified to include the dampening effect of the PDF (K_L) caused by 1/cos(θ)-dependent Lorentzian peak broadening in reciprocal space, as suggested by Beyer *et al.* (2022), since it is a common contribution to the peak shape for synchrotron diffraction data (Beyer *et al.*, 2022). Normally, only the Gaussian dampening effect (K_G, known as Q_{damp} in DiffPY-CMI) is included in modelling software. The modification to the modelling equation including both dampening terms becomes:

```
pdfcontribution.setEquation("(scale**2)**0.5 * (G - 4*3.1415926*r*rho0)
* exp(-1/2*(KG*r)**2/5.545177) * exp(-KL*r) * f(r,psize)
+ (scale_ph2**2)**0.5 * (G_ph2 - 4*3.1415926*r*rho0_ph2)
* exp(-1/2*(KG*r)**2/5.545177) * exp(-KL*r) * f_ph2(r,psize_ph2)")
```
(S2)

Here, G is the PDF calculated with the DSE from $Q_{min} = 0$, *f(r, psize)* corresponds to the dampening effect from monodisperse, spherical coherent domain sizes. Since the PDF is calculated to $Q_{min} = 0$, the $-4\pi r\rho_0$ term must be calculated explicitly and subtracted. Since it is unclear

how to access the average number density (ρ_0) within the DiffPy-CMI framework during a refinement, it is set based on the refined unit cell volume and occupancies as well as the number of atoms in the unit cell, according to the cubic phase:

recipe.constrain(pdfcontribution.rho0,
'4*((1-Y_occ) + Y_occ + (2-Y_occ/2))/(a*a*a)') (S3)

and according to for the monoclinic phase:

recipe.constrain(pdfcontribution.rho0_ph2,
'12 /(a_ph2*b_ph2*c_ph2*sin(be_ph2/180*3.1415926))') (S4)

S2. Supplementary figures

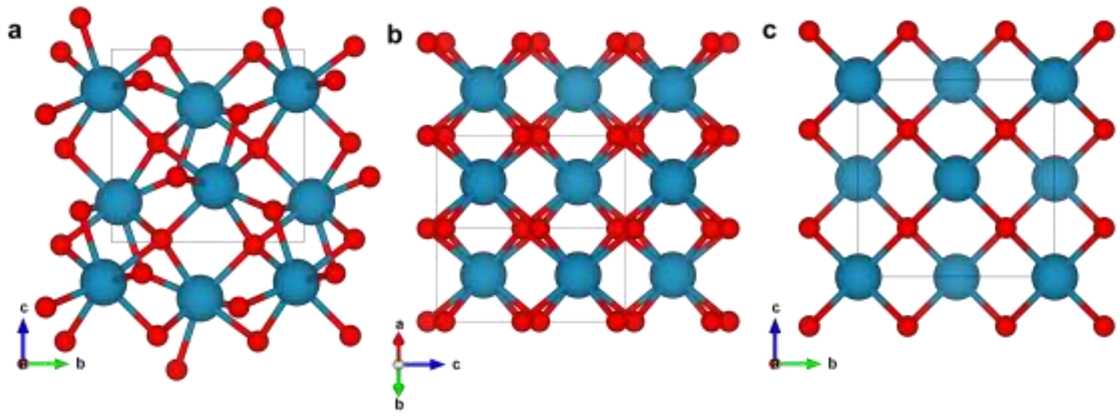

Figure S1 Crystal structures of HfO_2 polymorphs visualized to highlight their structural similarities. a) *m*-HfO_2 ($P2_1/c$) shown along [100] direction. b) *t*-HfO_2 ($P4_2/mnc$) shown along [110] direction. c) *c*-HfO_2 ($Fm\bar{3}m$) shown along [100] direction.

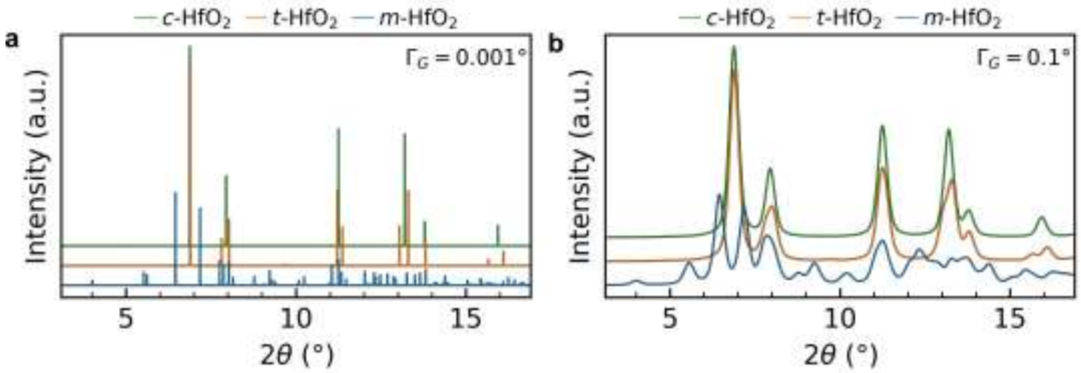

Figure S2 Calculated diffraction patterns of HfO_2 polymorphs simulated with a gaussian peak width (Γ_G) of a) 0.001° and b) 0.1°. The reference structures are: ICSD-142790 (*m*-HfO_2), ICSD-173966 (*t*-

HfO_2), and ICSD-53033 (c-HfO_2) with the unit cell parameters of t-HfO_2 (a = 3.5775 Å, c = 5.1996 Å) and c-HfO_2 (a = 5.111 Å).

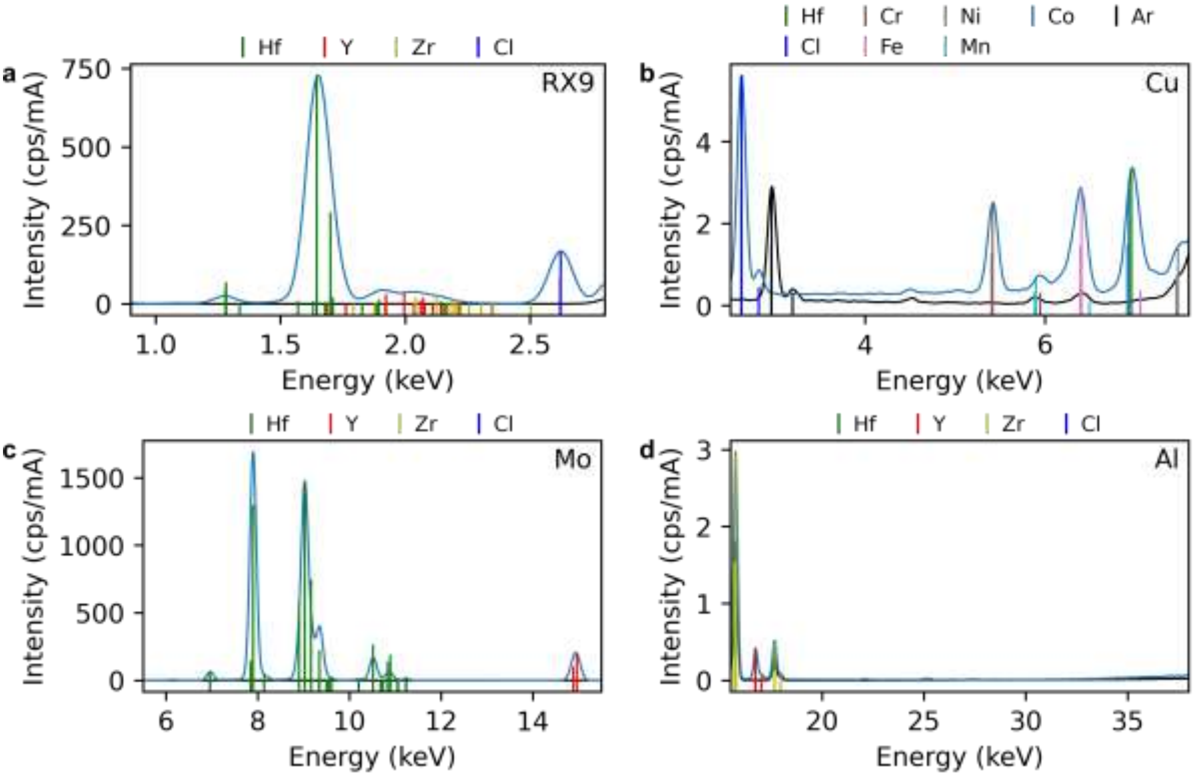

Figure S3 XRF spectra for as-prepared YSH8 sample together with a blank measurement (black) from different X-ray sources; (a) RX9, (b) Cu, (c) Mo, and (d) Al.

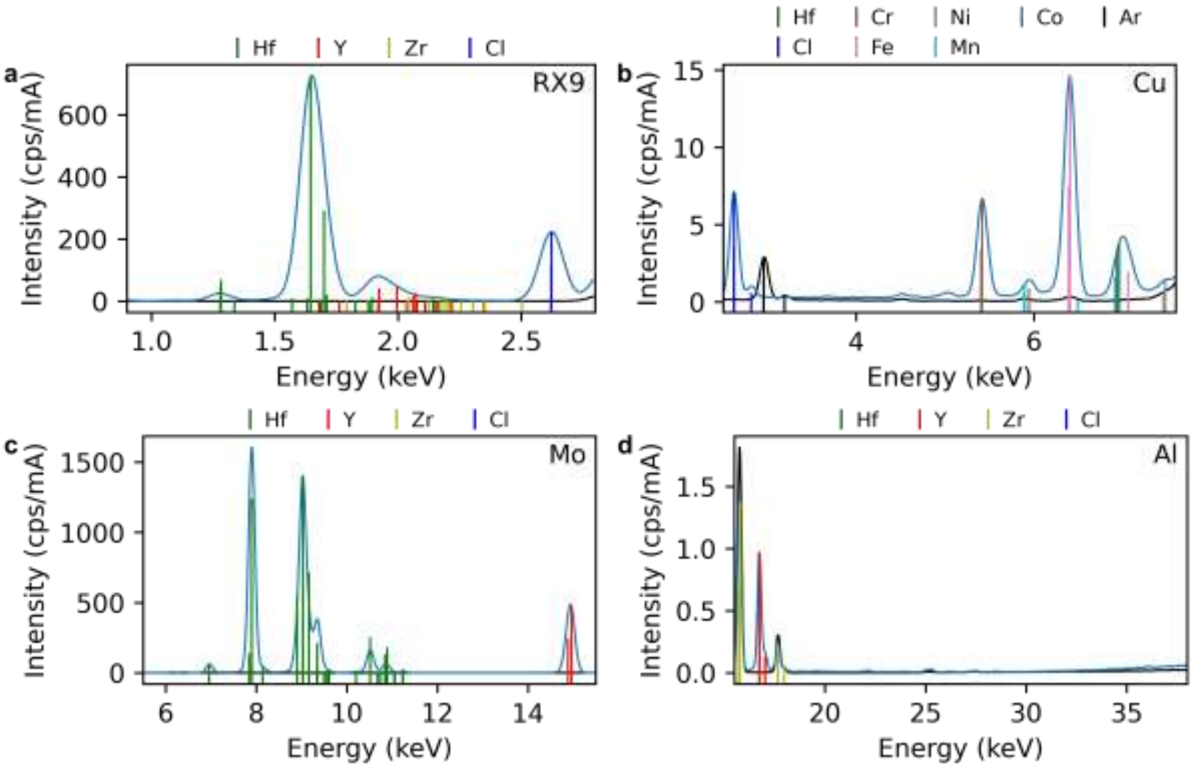

Figure S4 XRF spectra for as-prepared YSH16 sample together with a blank measurement (black) from different X-ray sources; (a) RX9, (b) Cu, (c) Mo, and (d) Al.

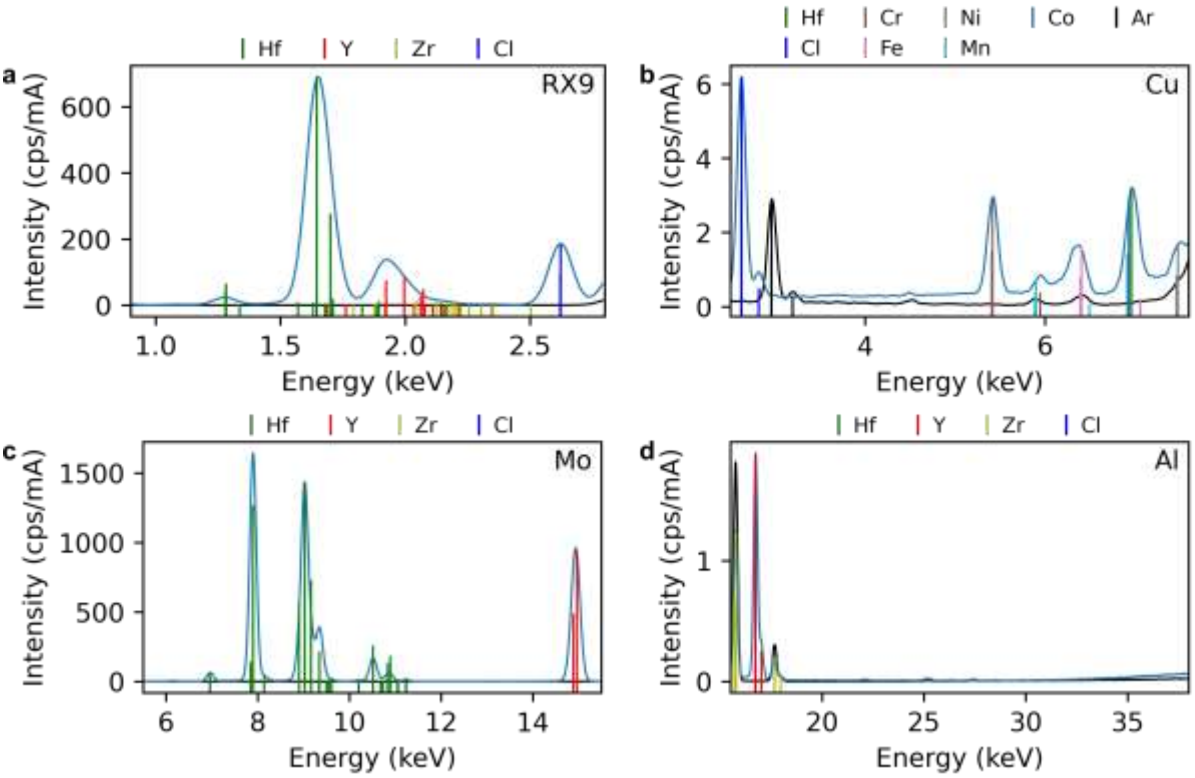

Figure S5 XRF spectra for as-prepared YSH32 sample together with a blank measurement (black) from different X-ray sources; (a) RX9, (b) Cu, (c) Mo, and (d) Al.

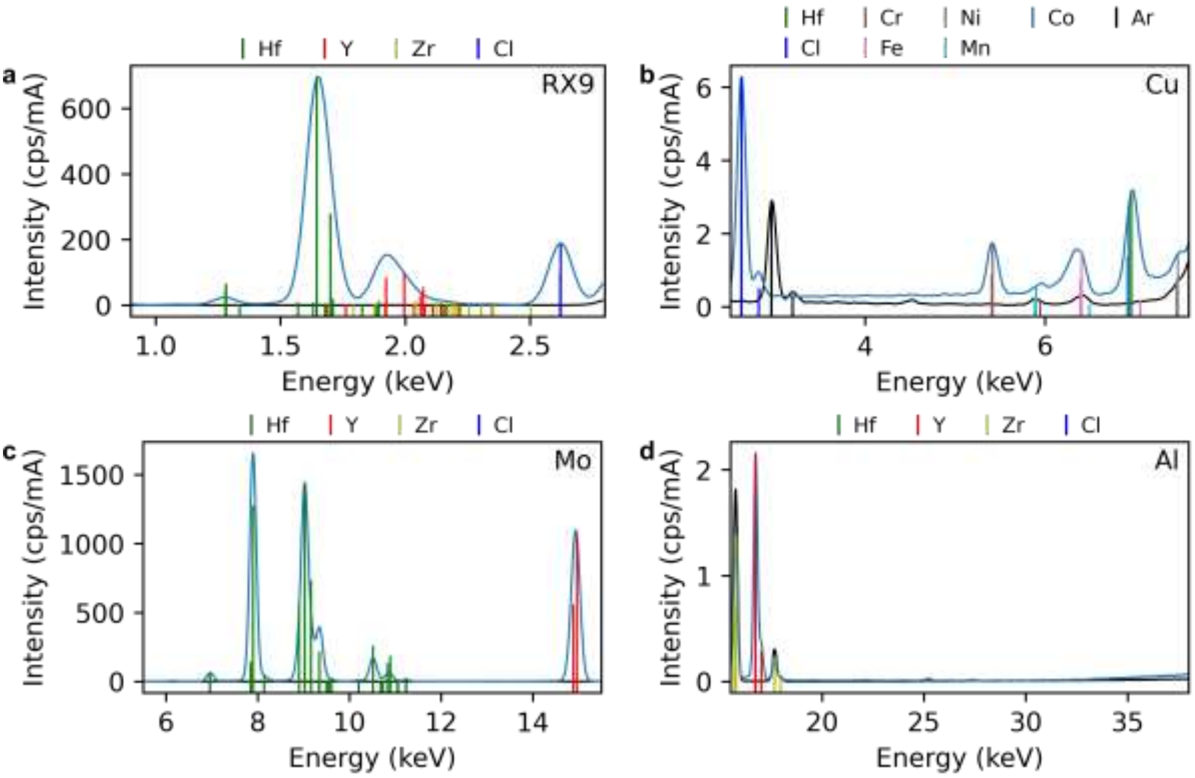

Figure S6 XRF spectra for as-prepared YSH48 sample together with a blank measurement (black) from different X-ray sources; (a) RX9, (b) Cu, (c) Mo, and (d) Al.

Table S1 XRF results of as-prepared YSH samples. ND: not detected. NQ: not quantified.

	Hf (at%)	Y (at%)	Zr (at%)	Cl (at%)	Cr (at%)	Mn (at%)	Fe (at%)	Co (at%)	Ni (at%)
YSH8	86.76(13)	5.02(9)	NQ	7.98(4)	0.135(5)	ND	0.100(7)	ND	ND
YSH16	77.65(6)	11.48(5)	NQ	10.08(5)	0.362(2)	ND	0.455(8)	ND	ND
YSH32	71.78(4)	20.22(4)	NQ	7.81(2)	0.142(2)	ND	0.072(6)	ND	ND
YSH48	70.06(9)	22.08(4)	NQ	7.74(3)	0.0799(14)	ND	0.061(17)	ND	ND

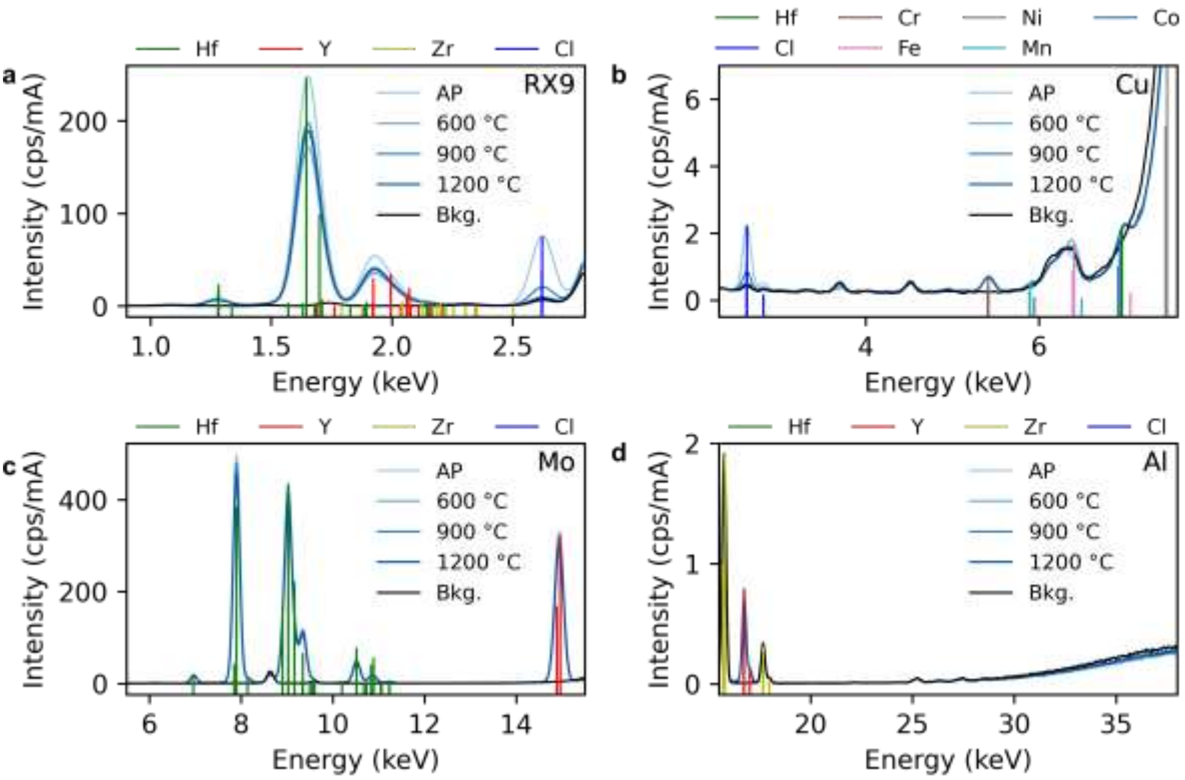

Figure S7 XRF spectra for YSH48 sample, both as-prepared and samples annealed at 600-1200 °C together with a blank measurement (black), from different X-ray sources; (a) RX9, (b) Cu, (c) Mo, and (d) Al.

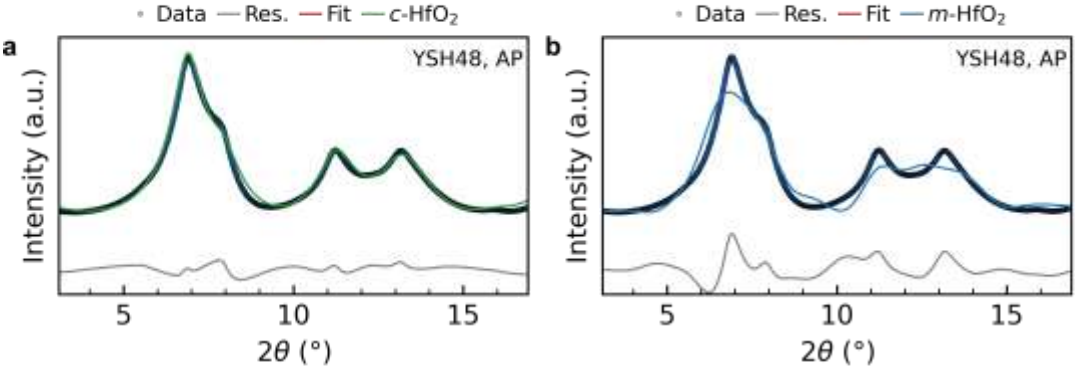

Figure S8 PXRD pattern of as-prepared YSH48 sample modelled with (a) *c*-HfO_2 phase and (b) *m*-HfO_2 phase.

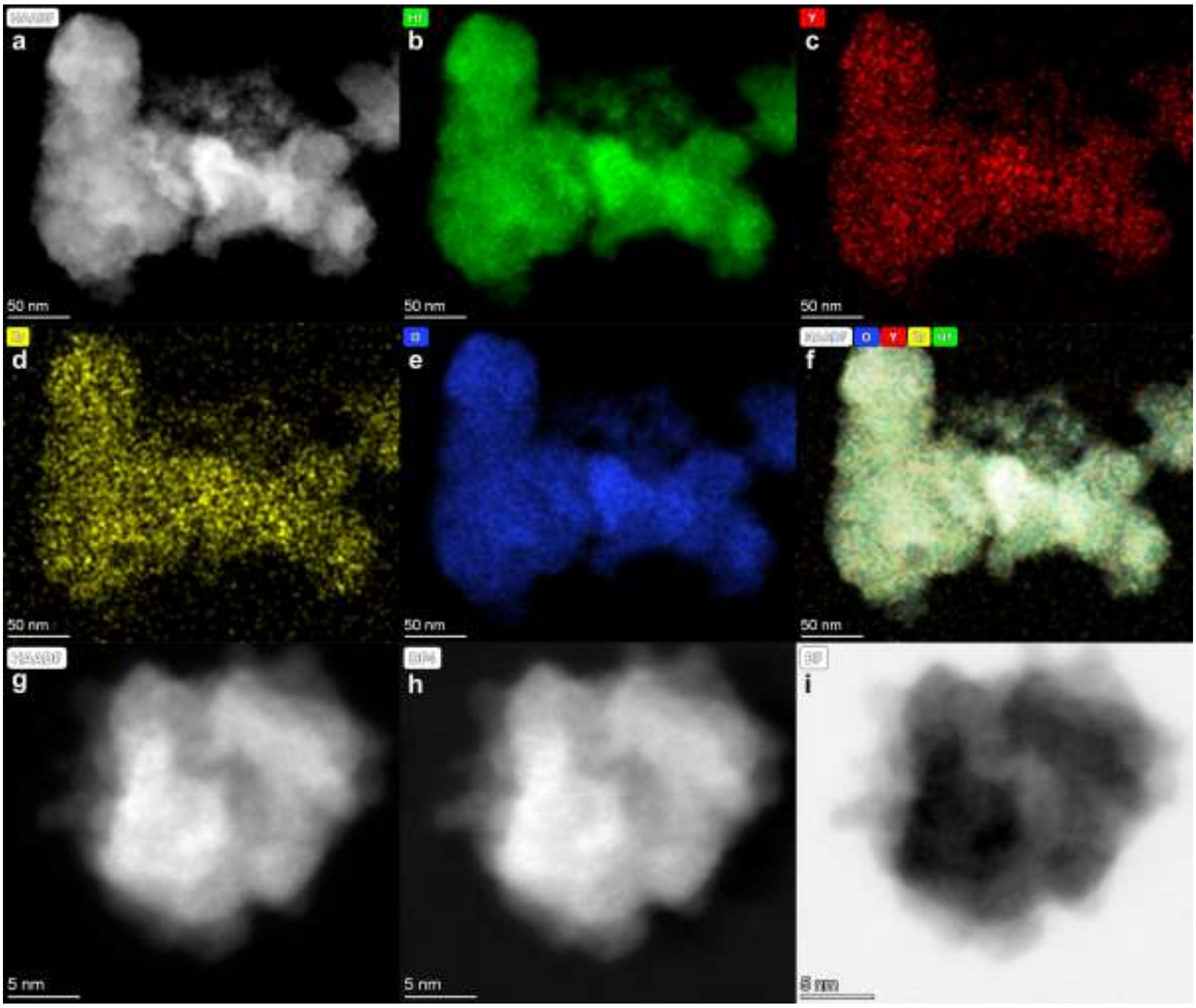

Figure S9 STEM-EDX images of as-prepared YSH8 sample. (a) HAADF image of larger particle agglomerate and corresponding EDX maps of (b) Hf, (c) Y, (d) Zr, (e) O, and (f) combined overlay of (a-e). (g) HAADF, (g) dark-field, and (i) bright-field image of a smaller particle.

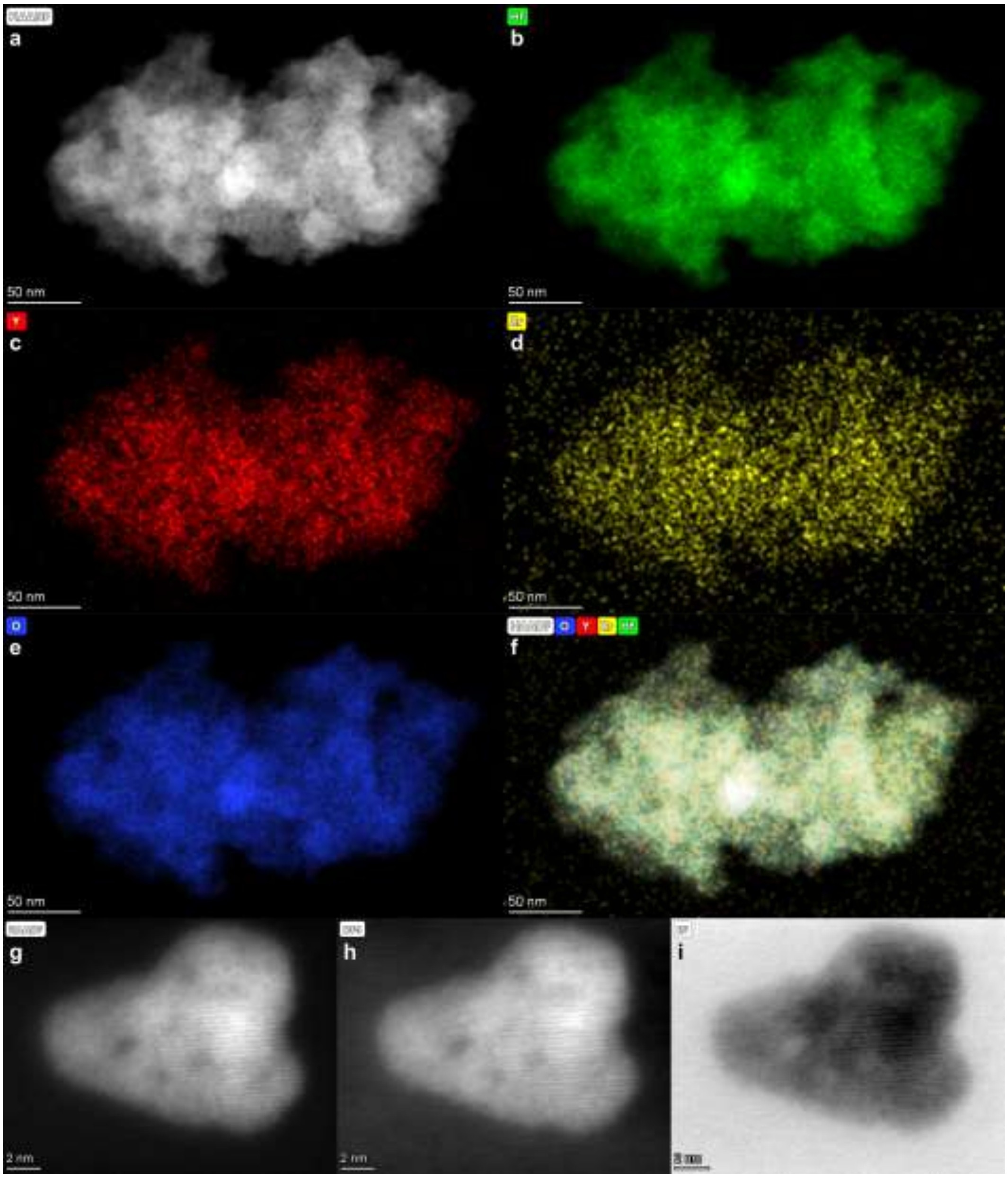

Figure S10 STEM-EDX images of as-prepared YSH16 sample. (a) HAADF image of larger particle agglomerate and corresponding EDX maps of (b) Hf, (c) Y, (d) Zr, (e) O, and (f) combined overlay of (a-e). (g) HAADF, (g) dark-field, and (i) bright-field image of a smaller particle.

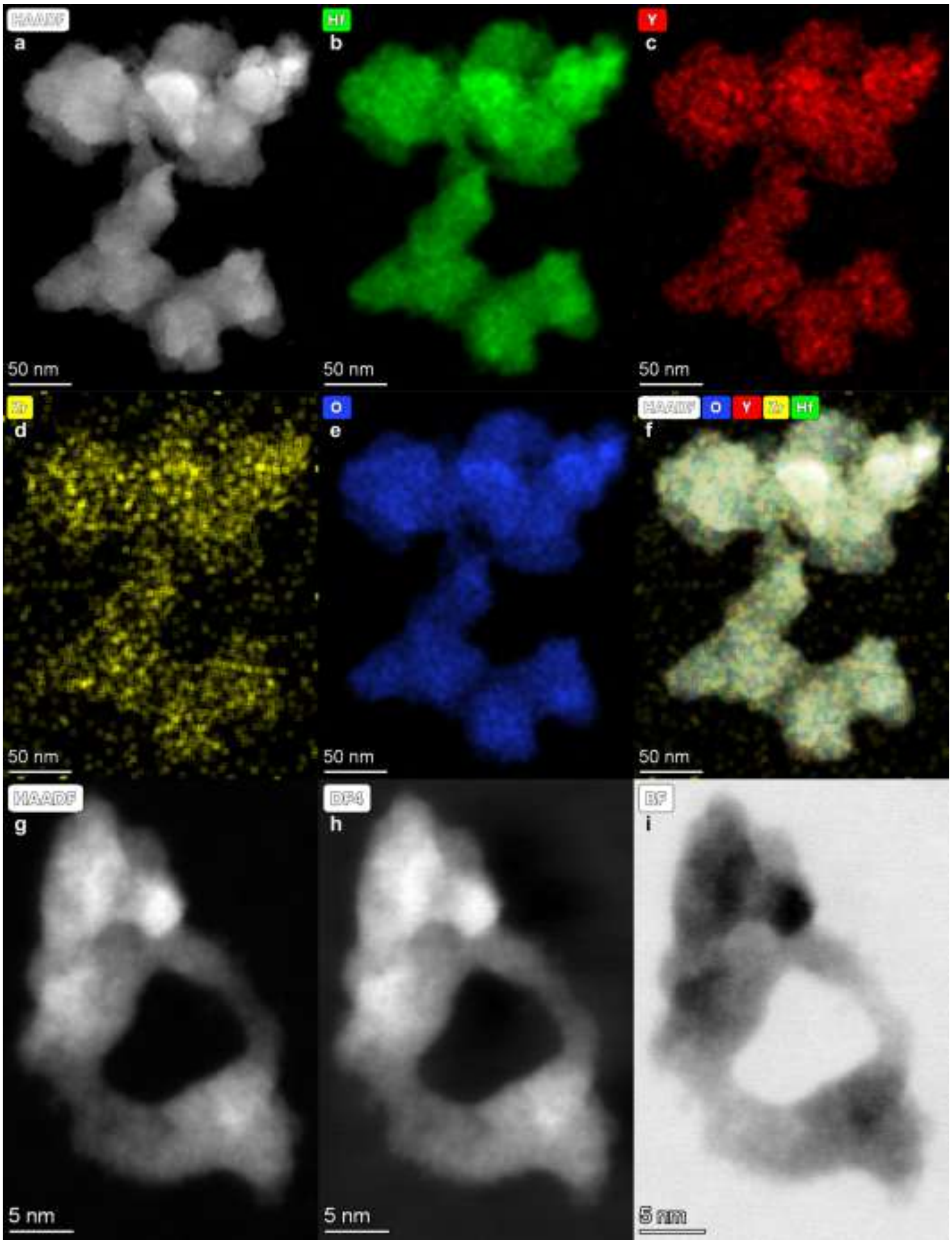

Figure S11 STEM-EDX images of as-prepared YSH32 sample. (a) HAADF image of larger particle agglomerate and corresponding EDX maps of (b) Hf, (c) Y, (d) Zr, (e) O, and (f) combined overlay of (a-e). (g) HAADF, (g) dark-field, and (i) bright-field image of a smaller particle.

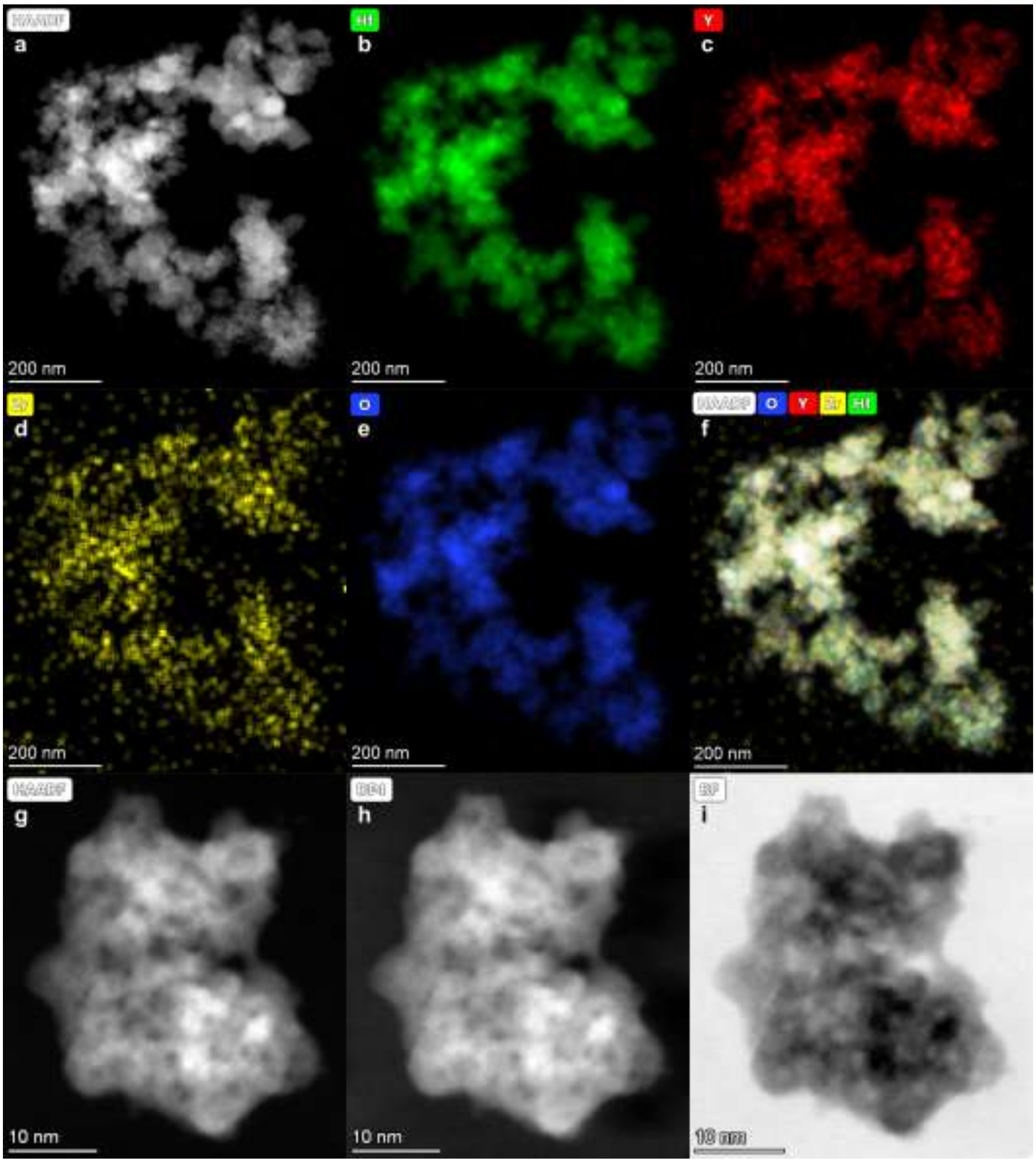

Figure S12 STEM-EDX images of as-prepared YSH48 sample. (a) HAADF image of larger particle agglomerate and corresponding EDX maps of (b) Hf, (c) Y, (d) Zr, (e) O, and (f) combined overlay of (a-e). (g) HAADF, (g) dark-field, and (i) bright-field image of a smaller particle.

Table S2 Experimental determination of the actual Y^{3+} content in the YSH samples compared to the nominal composition, as determined by ICP-OES, XRF, and STEM-EDX. For XRF and STEM-EDX, Zr has not been quantified since the instruments themselves produce Zr impurity signals. NQ: not quantified.

Sample	Nominal (at%)			ICP-OES (at%)			XRF (at%)			STEM-EDX (at%)		
	Hf	Zr	Y	Hf	Zr	Y	Hf	Zr	Y	Hf	Zr	Y
YSH8	92	-	8	90.39(5)	4.30(3)	5.31(3)	94.53(9)	NQ	5.47(9)	94.8(7)	NQ	5.2(7)
YSH16	84	-	16	86.73(7)	0.46(5)	12.81(5)	87.09(6)	NQ	12.91(6)	85.3(18)	NQ	14.7(18)
YSH32	68	-	32	77.2(2)	0.467(19)	22.3(2)	78.02(4)	NQ	21.98(4)	87.0(17)	NQ	13.0(17)
YSH48	52	-	48	75.0(3)	0.49(5)	24.5(3)	76.04(4)	NQ	23.96(4)	75(3)	NQ	25(3)

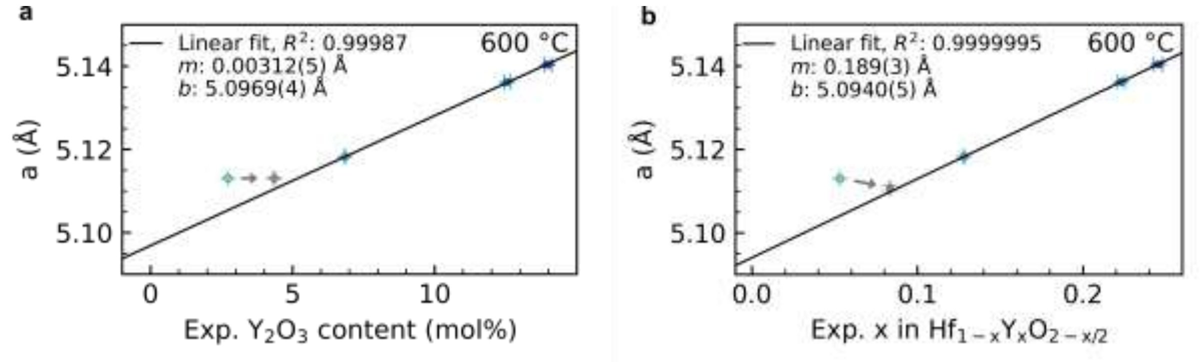

Figure S13 (a) Refined unit cell parameters of the *c*-HfO_2 phase *versus* experimental mol% Y_2O_3 as determined from ICP-OES. Black line corresponds to fit from orthogonal distance linear regression to the unit cell parameters of the phase-pure samples. The grey point corresponds to the unit cell parameter of YHS8 moved horizontally on the composition axis by dividing the ICP-OES composition with the refined molar fraction of the *c*-HfO_2 phase. (b) Refined unit cell parameters of *c*-HfO_2 phase *versus* experimental Y^{3+} content, x, as determined from ICP-OES. YSH8 moved horizontally on the composition axis by dividing ICP-OES composition with refined molar fraction of *c*-HfO_2 phase and vertically *via* Vegards law between ZrO_2 and HfO_2 based on Zr-content as determined by ICP-OES. Used lattice parameter of *c*-ZrO_2 is 5.120 Å (Kim, 1989).

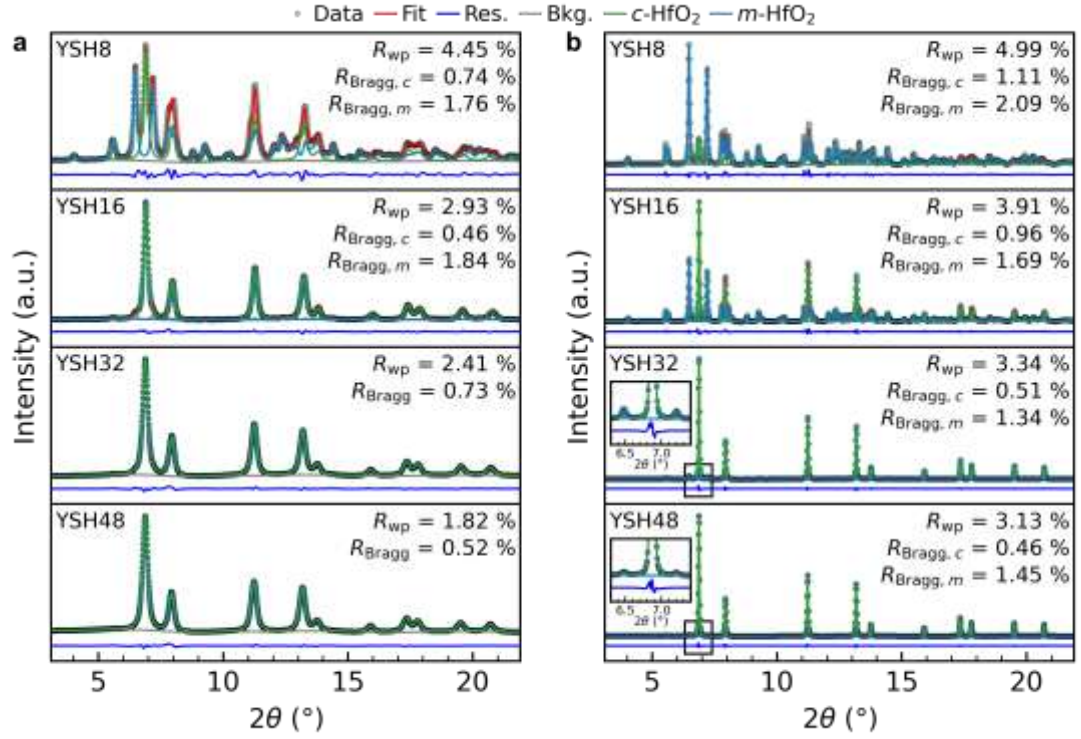

Figure S14 Refined PXRD patterns of YSH samples annealed at (a) 900 °C and (b) 1200 °C.

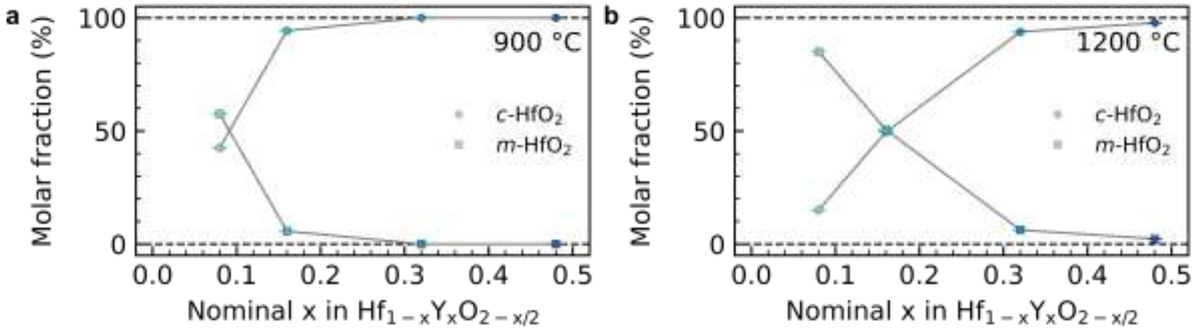

Figure S15 Refined molar fractions against nominal composition for YSH samples annealed at (a) 900 °C and (b) 1200 °C.

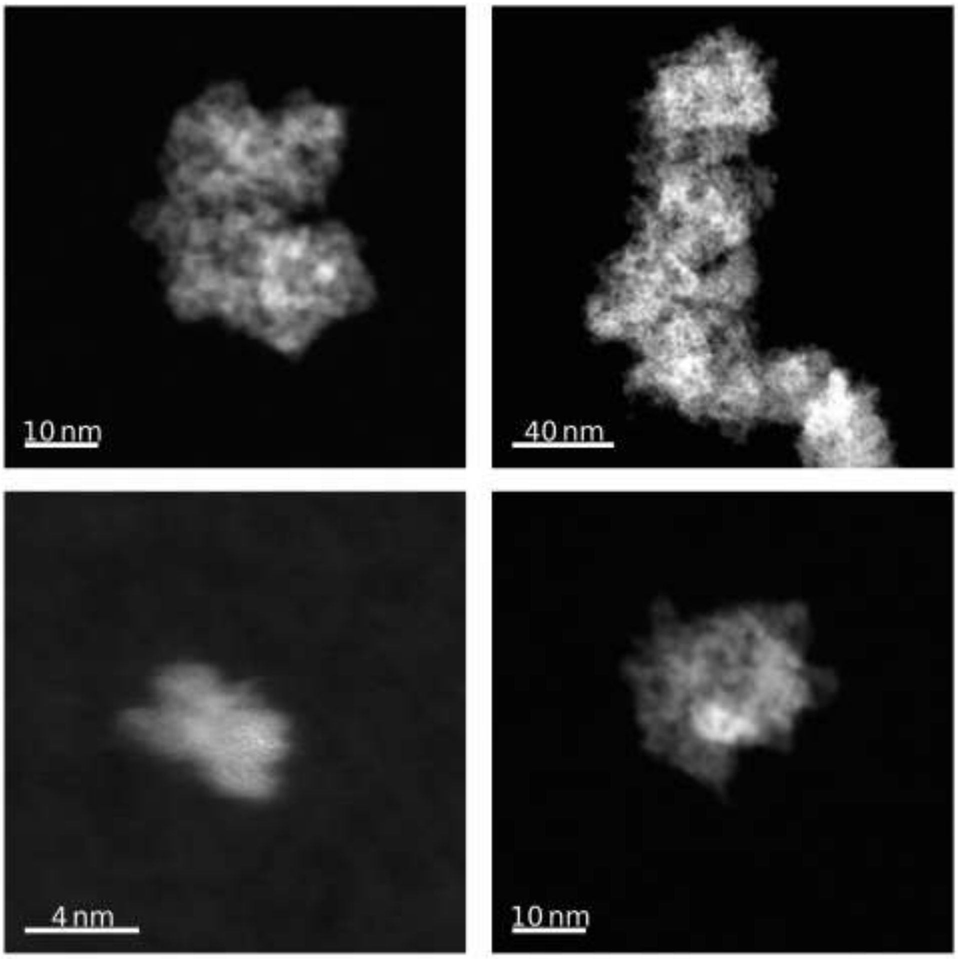

Figure S16 STEM (HAADF) images of YSH48 sample as-prepared.

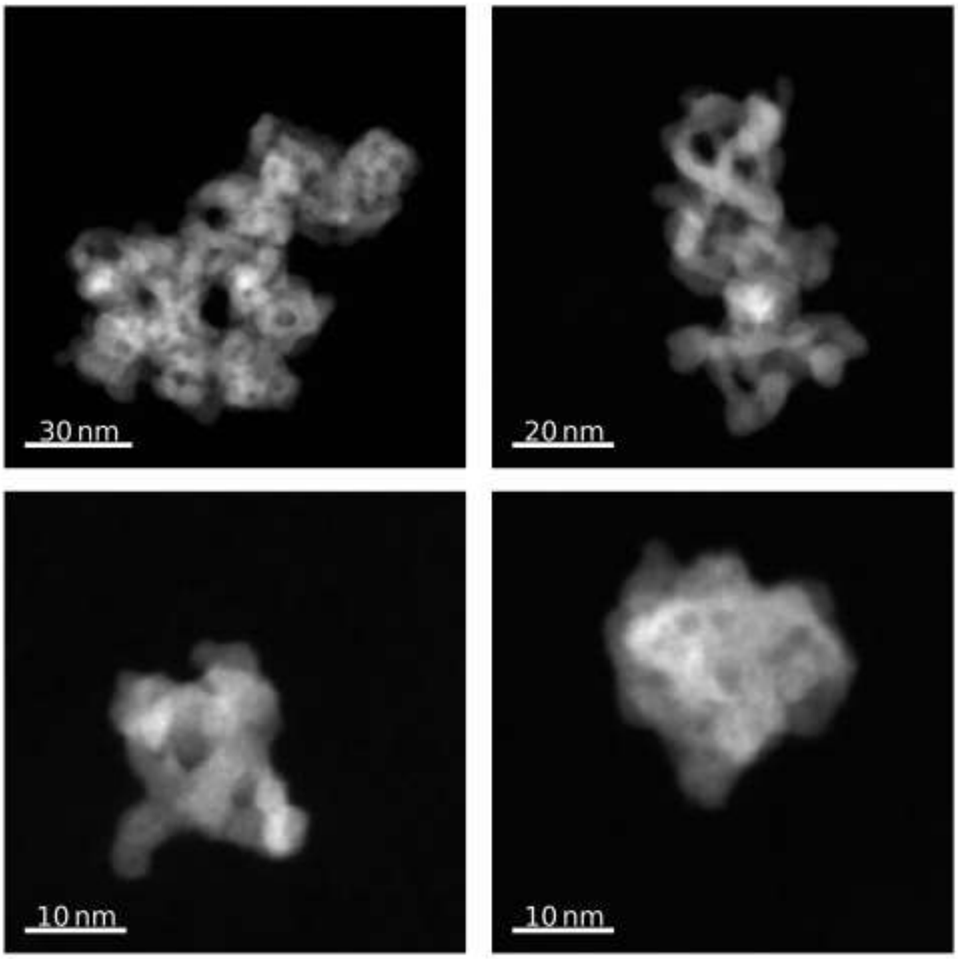

Figure S17 STEM (HAADF) images of YSH48 sample annealed at 600 °C.

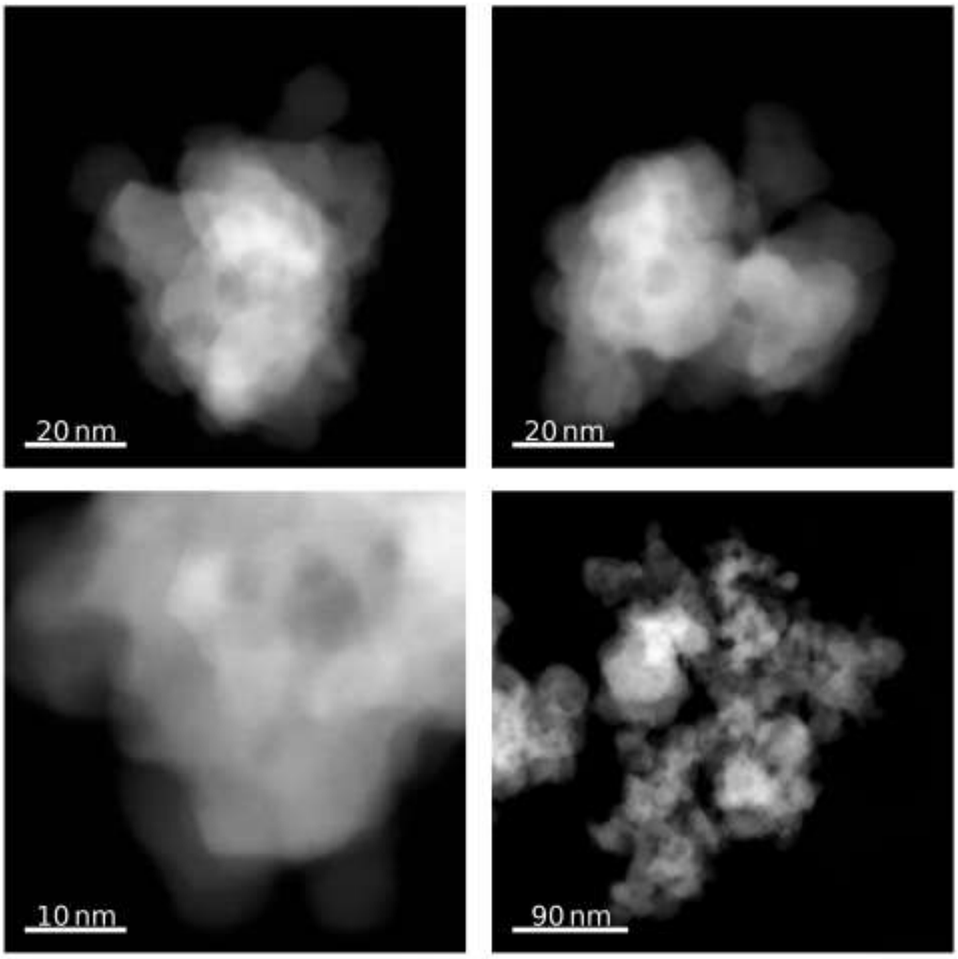

Figure S18 STEM (HAADF) images of YSH48 sample annealed at 900 °C.

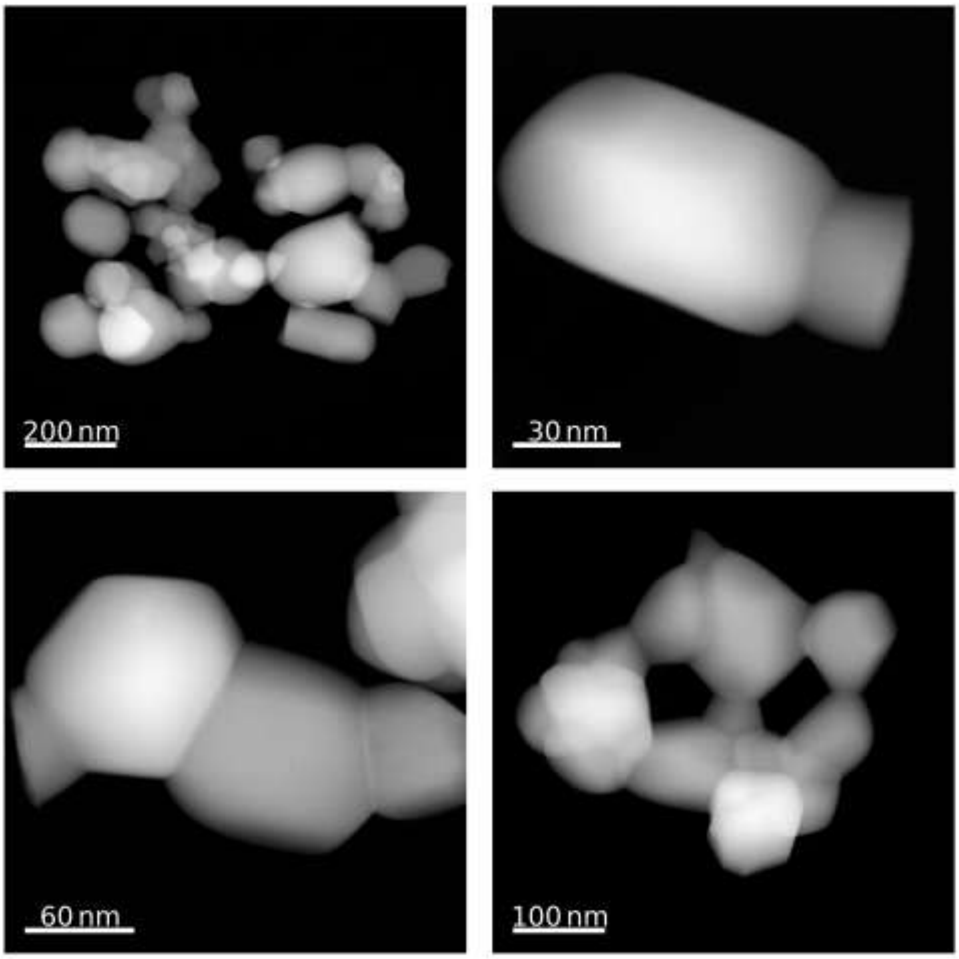

Figure S19 STEM (HAADF) images of YSH48 sample annealed at 1200 °C.

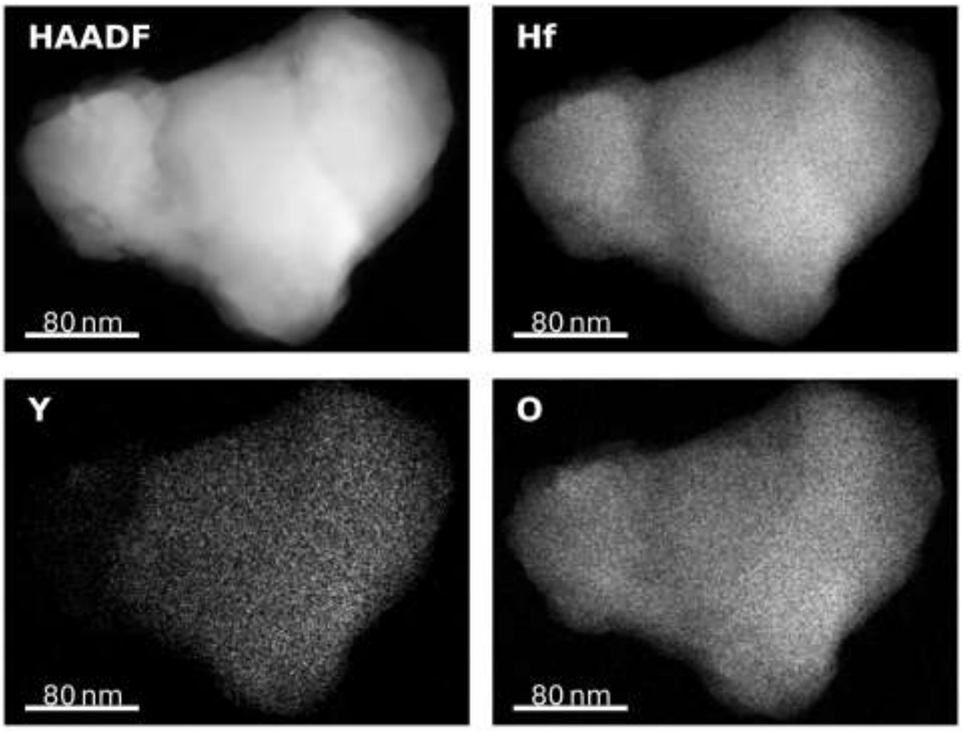

Figure S20 Elemental maps and HAADF image obtained with STEM-EDX of YSH16 sample annealed at 1200 °C. The Y-rich region has an estimated average composition of $Hf_{0.96(7)}Y_{0.27(3)}O_{2.00(7)}$, and the Y-poor region has an estimated average composition of $Hf_{0.96(7)}Y_{0.056(7)}O_{2.00(7)}$.

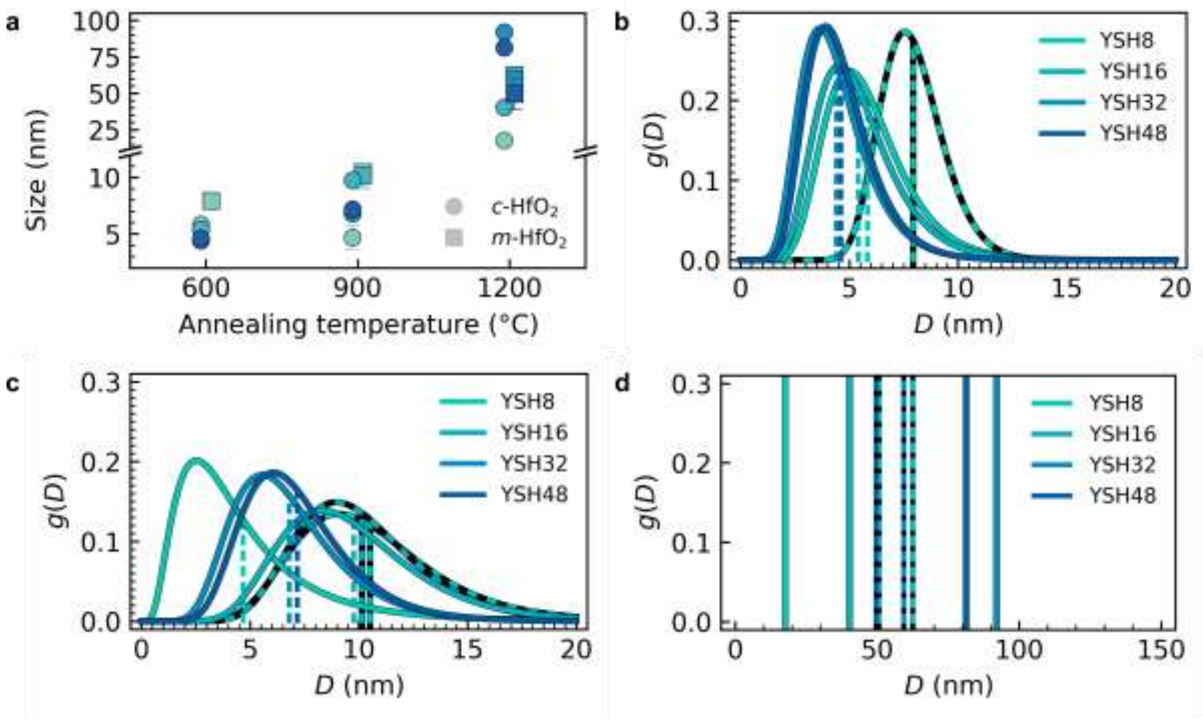

Figure S21 (a) Mean sizes extracted from lognormal WPPM of the PXRD patterns for both c-HfO_2 (○) and m-HfO_2 (□) phases, calculated according to Scardi (2020) and (b-d) corresponding lognormal size distributions for samples annealed at (b) 600 °C, (c) 900 °C, and (d) 1200 °C. Black-dashed curves correspond to m-HfO_2 phase and are only included for two-phase samples. Vertical lines correspond to mean sizes of the distributions. For samples annealed at 1200 °C, only vertical lines are shown to highlight that these were modelled assuming monodisperse spherical particles.

Table S3 Mean sizes and distribution widths from lognormal WPPM of PXRD patterns.

**Sample**	**600 °C**				**900 °C**				**1200 °C**			
	c-HfO_2		m-HfO_2		c-HfO_2		m-HfO_2		c-HfO_2		m-HfO_2	
	$\langle D\rangle_c$	std_c	$\langle D\rangle_m$	std_m	$\langle D\rangle_c$	std_c	$\langle D\rangle_m$	std_m	$\langle D\rangle_c$	std_c	$\langle D\rangle_m$	std_m
YSH8	5.8(2)	3.73(16)	7.93(9)	4.89(5)	5.7(10)	3.5(8)	10.5(4)	6.7(2)	17.58(13)	-	50.6(7)	-
YSH16	5.41(10)	3.48(7)	-	-	9.8(2)	6.25(16)	10.1(11)	6.4(7)	40.2(2)	-	62.5(16)	-
YSH32	4.40(9)	2.84(6)	-	-	6.80(12)	4.41(8)	-	-	92.0(3)	-	59(4)	-
YSH48	4.61(8)	2.95(5)	-	-	7.18(10)	4.60(6)	-	-	81.3(2)	-	50(10)	-

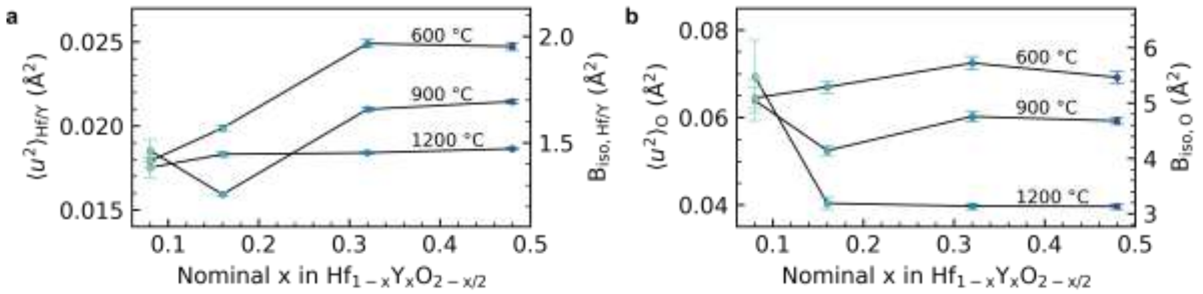

Figure S22 Refined atomic displacement parameters (ADPs) of *c*-HfO_2 phase from PXRD refinements for (a) metal site and (b) oxygen site.

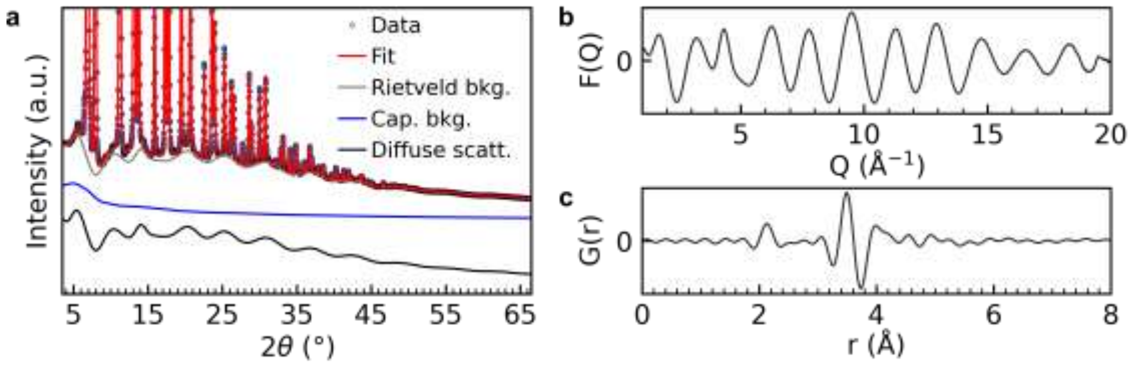

Figure S23 Diffuse scattering in YSH samples. (a) Zoom-in of Rietveld refinement of YSH48 sample annealed at 1200 °C, highlighting the diffuse scattering contribution to the PXRD pattern. The modelled background used in the refinement (grey) is comprised of a measurement of an empty capillary (blue) together with a multi-order Chebyshev polynomial and several broad Gaussian functions to account for the diffuse scattering contribution (black). (b) Reduced structure function, F(Q), obtained from the diffuse scattering contribution in (a). (c) PDF of the diffuse scattering contribution, which is the same as that shown in Figure 3b (bottom).

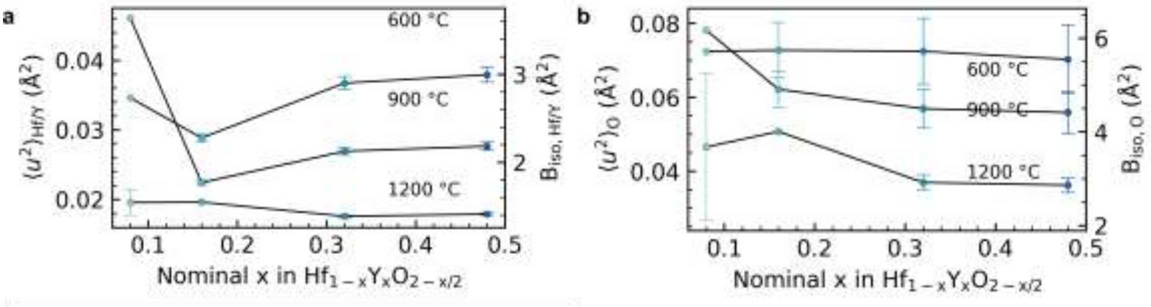

Figure S24 Refined ADPs of *c*-HfO_2 phase from PDF refinements for (a) metal site and (b) oxygen site.

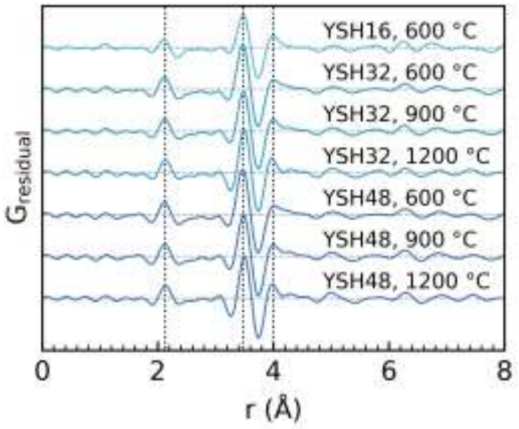

Figure S25 Comparison of PDF residual curves from 0-50 Å fits of phase-pure samples.

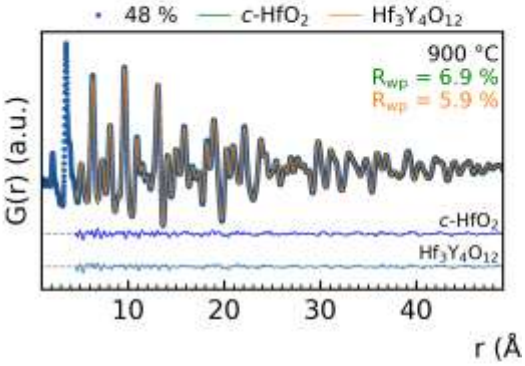

Figure S26 Comparison of modelling between 4.5 and 50 Å of *c*-HfO_2 and $Hf_3Y_4O_{12}$ phases.

Table S4 Structural parameters from fits used in Figure 4 and S26.

Fitting range	1–50 Å		4.5–50 Å	
**Phase**	***c*-HfO_2**	**$Hf_3Y_4O_{12}$**	***c*-HfO_2**	**$Hf_3Y_4O_{12}$**
a	5.1384(6)	9.590(4)	5.1394(6)	9.588(4)
c	-	8.951(7)	-	8.962(9)
Hf_{Biso}	2.18(5)	1.56(5)	2.29(6)	1.93(9)
O_{Biso}	4.4(4)	1.5(6)	6.0(7)	2.3(7)
δ_1	0.8(1)	2.36(7)	1.4(2)	1.4(3)
r (nm)	6.8(1)	7.3(2)	7.1(1)	7.4 (2)
Hf_x	-	0.1291(7)	-	0.142(3)
Hf_y	-	0.4140(7)	-	0.426(3)
Hf_z	-	0.0134(7)	-	0.012(1)

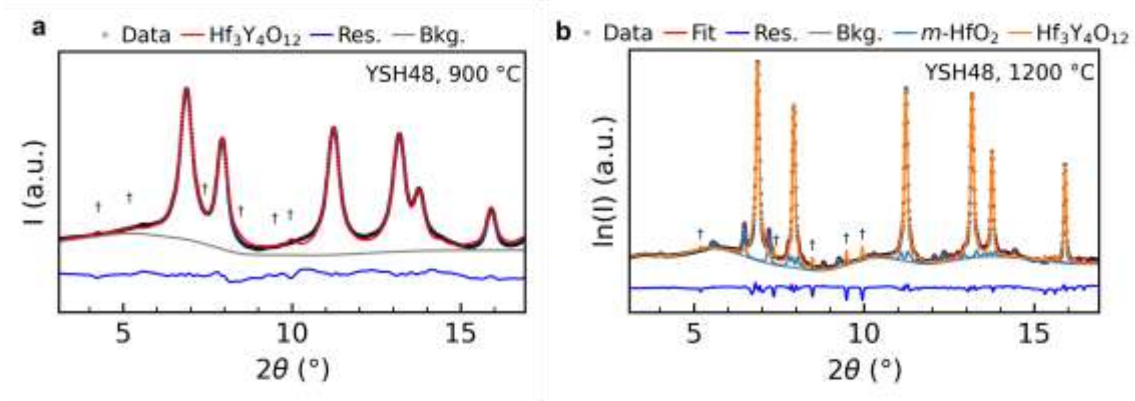

Figure S27 Rietveld refinement of PXRD pattern of YSH48 sample annealed at (a) 900 °C and (b) 1200 °C with the $Hf_3Y_4O_{12}$ phase (δ-phase) refining apart from the unit cell parameters, peak broadening, and ADPs also the position of the 18*f* cation site. In (b) the data and fit are shown on a logarithmic scale to highlight the additional peaks dictated by the rhombohedral symmetry of the δ-phase which does not appear in the experimental PXRD pattern.

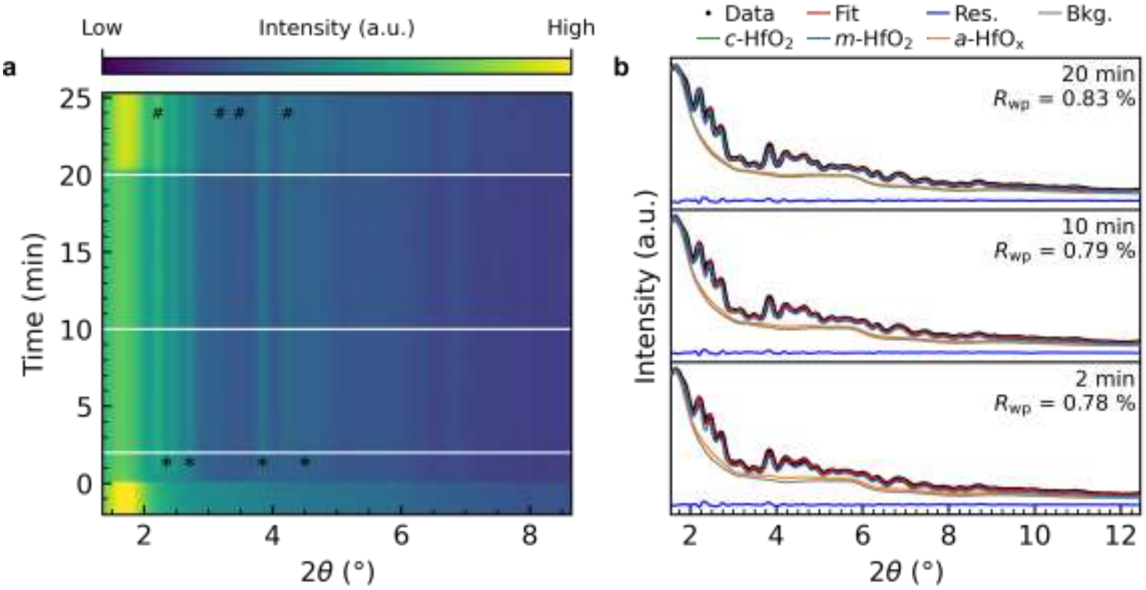

Figure S28 (a) 2D contour plot of unmodelled PXRD patterns during the *in situ* experiment of x = 0.00. (b) Selected Rietveld refinements of PXRD patterns during the experiment (2, 10, and 20 min).

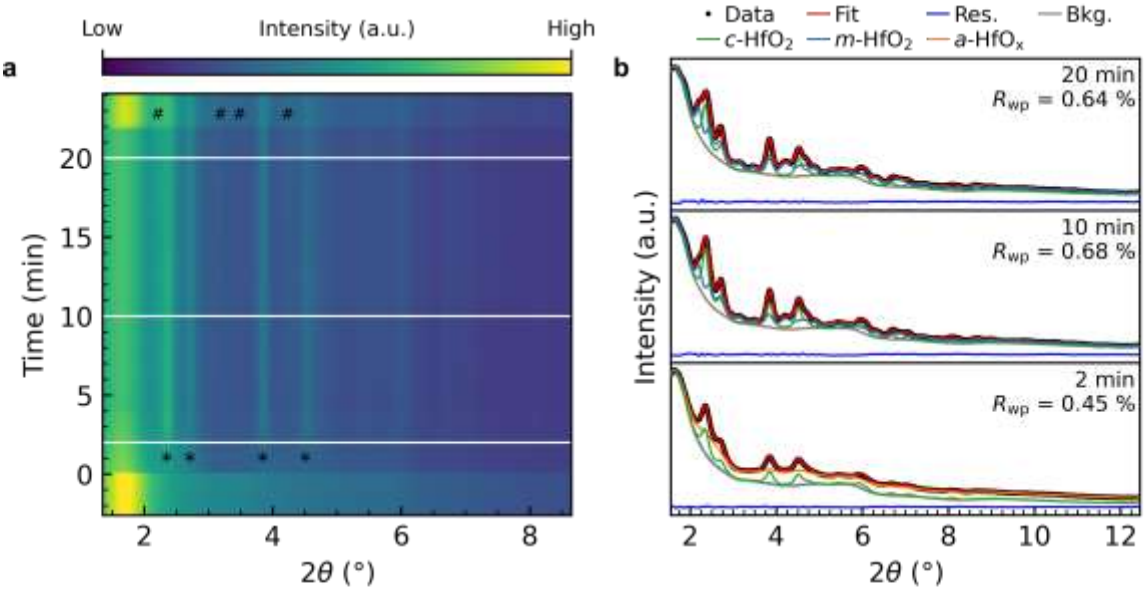

Figure S29 (a) 2D contour plot of unmodelled PXRD patterns during the *in situ* experiment of x = 0.08. (b) Selected Rietveld refinements of PXRD patterns during the experiment (2, 10, and 20 min).

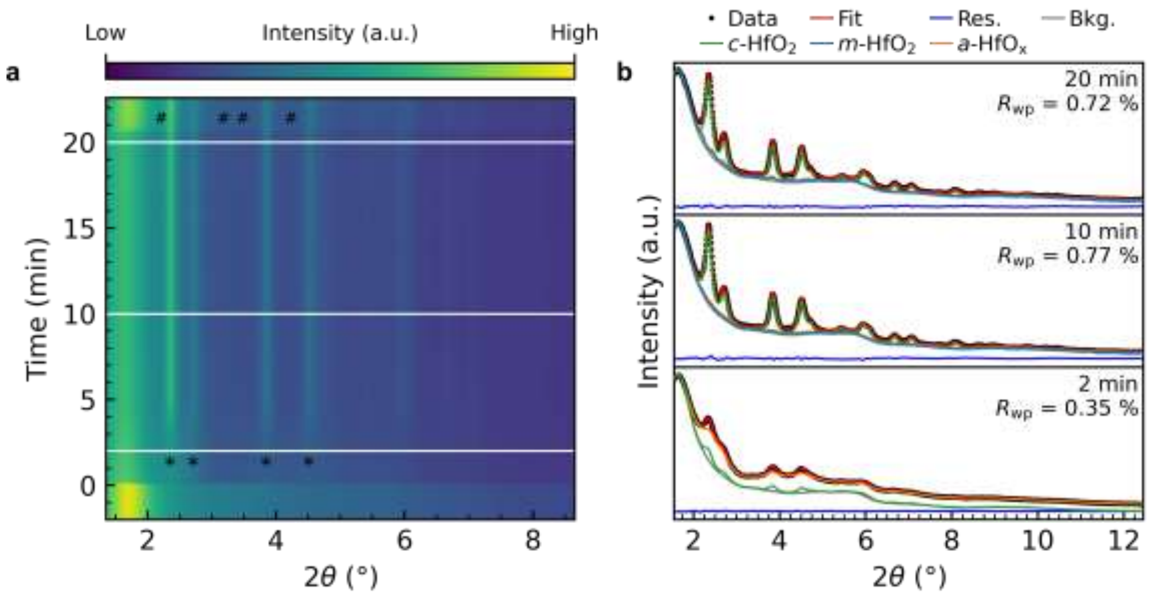

Figure S30 (a) 2D contour plot of unmodelled PXRD patterns during the *in situ* experiment of x = 0.16. (b) Selected Rietveld refinements of PXRD patterns during the experiment (2, 10, and 20 min).

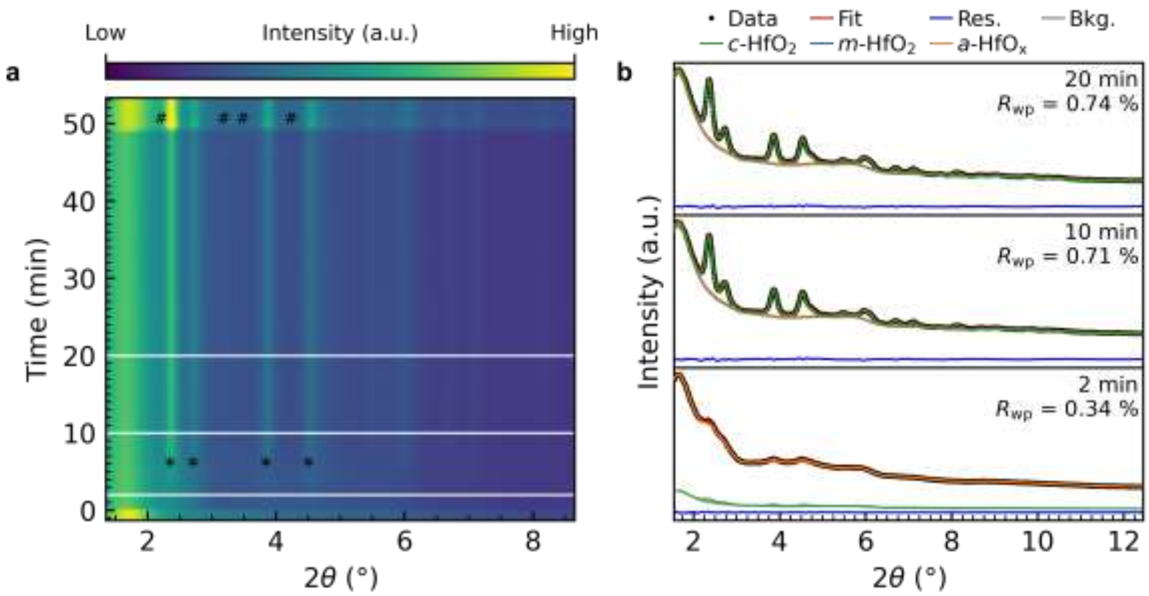

Figure S31 (a) 2D contour plot of unmodelled PXRD patterns during the *in situ* experiment of x = 0.24. (b) Selected Rietveld refinements of PXRD patterns during the experiment (2, 10, and 20 min).

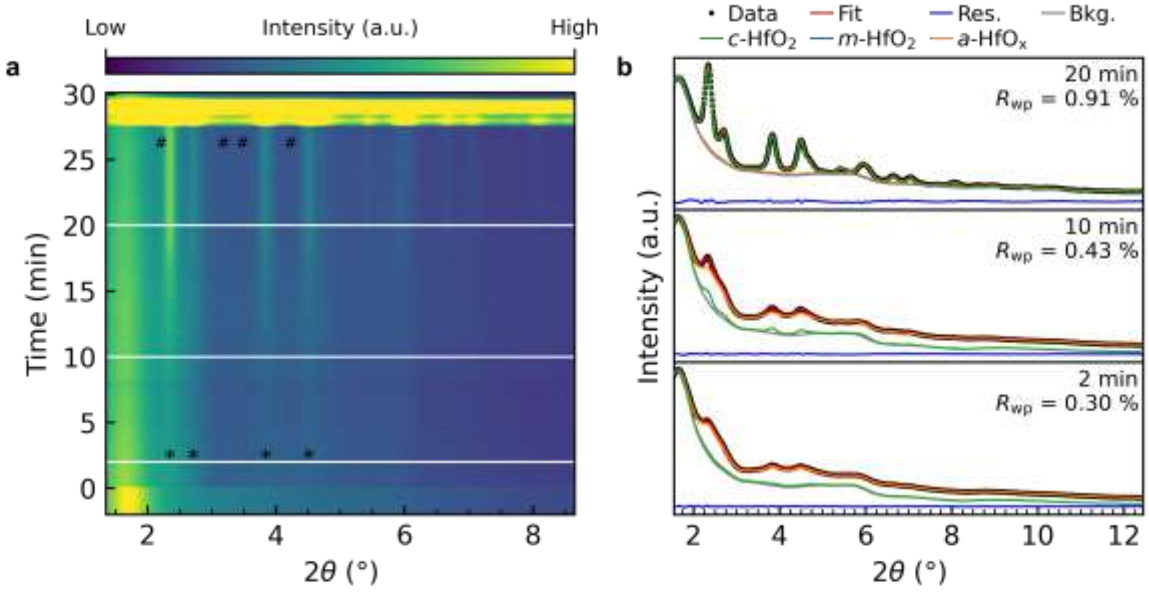

Figure S32 (a) 2D contour plot of unmodelled PXRD patterns during the *in situ* experiment of x = 0.32. (b) Selected Rietveld refinements of PXRD patterns during the experiment (2, 10, and 20 min).

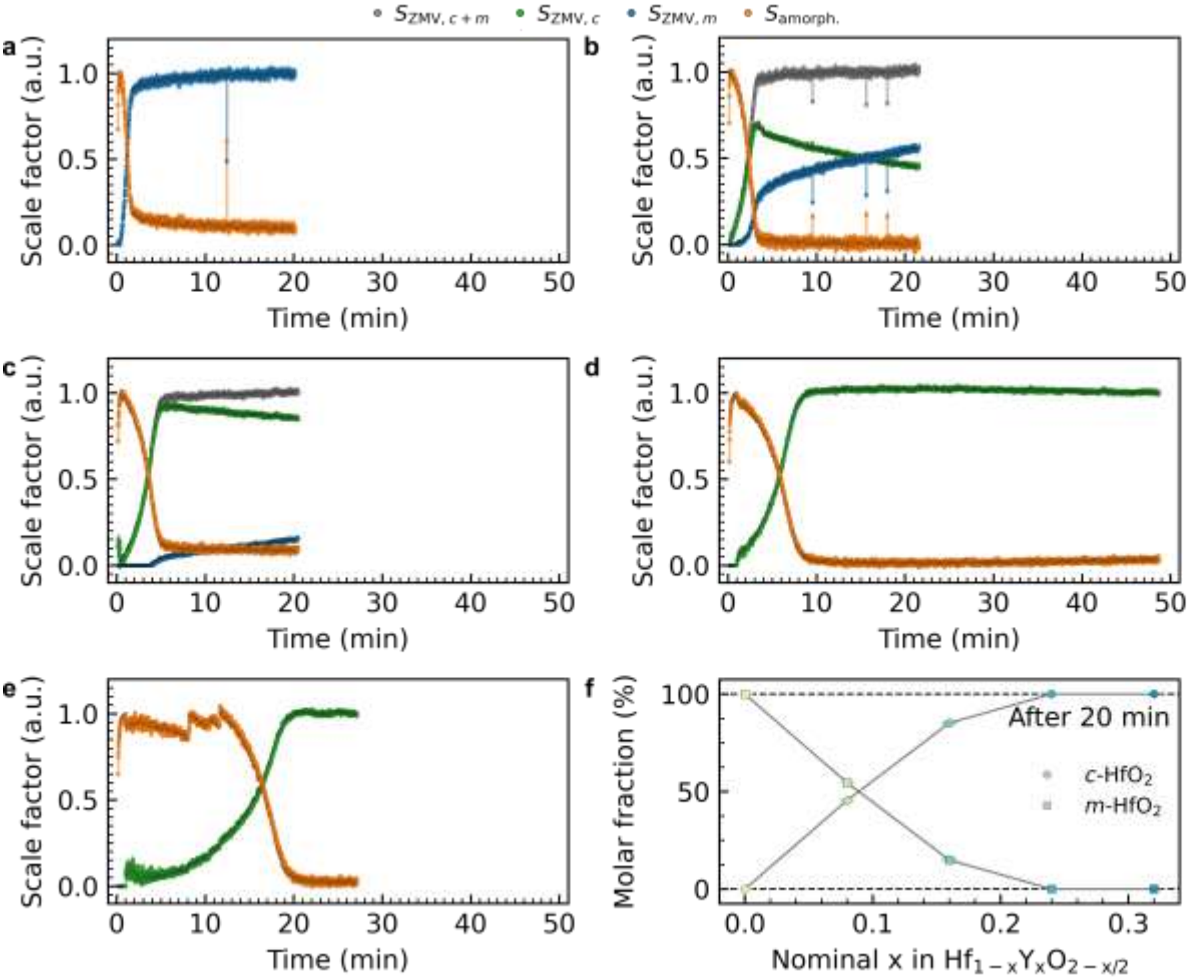

Figure S33 (a-e) ZMV-weighted scale factors for *c*-HfO_2 (green), *m*-HfO_2 (blue), and the sum of *c*-HfO_2 and *m*-HfO_2 (grey) phases together with scale factor of the amorphous contribution (orange) for (a) x = 0.00, (b) x = 0.08, (c) x = 0.16, (d) x = 0.24, and (e) x = 0.32. (f) Refined molar fractions of *c*-HfO_2 and *m*-HfO_2 after 20 min of synthesis plotted against nominal composition.

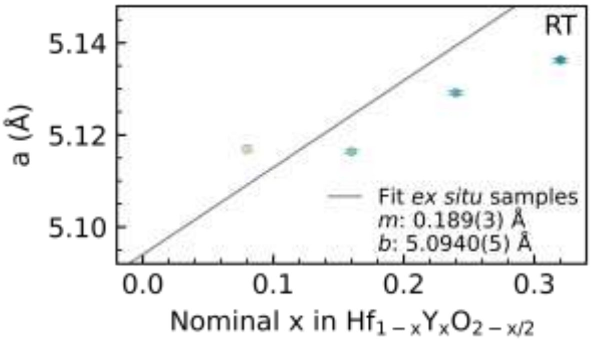

Figure S34 Refined unit cell parameters of *c*-HfO_2 after synthesis and cooling to room temperature plotted against nominal composition.

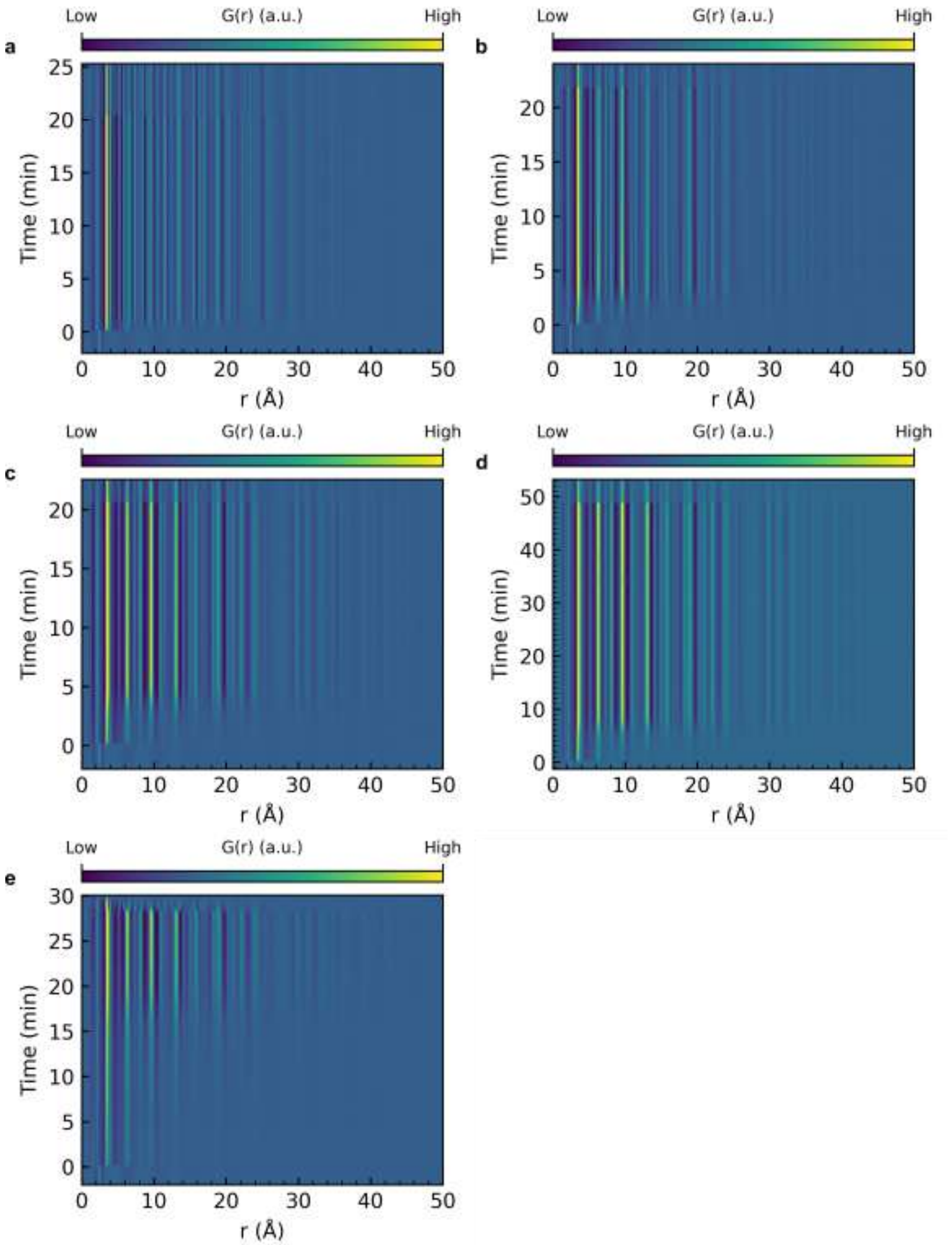

Figure S35 2D contour plot of unmodelled PDFs during the *in situ* experiment of (a) x = 0.00, (b) x = 0.08, (c) x = 0.16, (d) x = 0.24, and (e) x = 0.32.

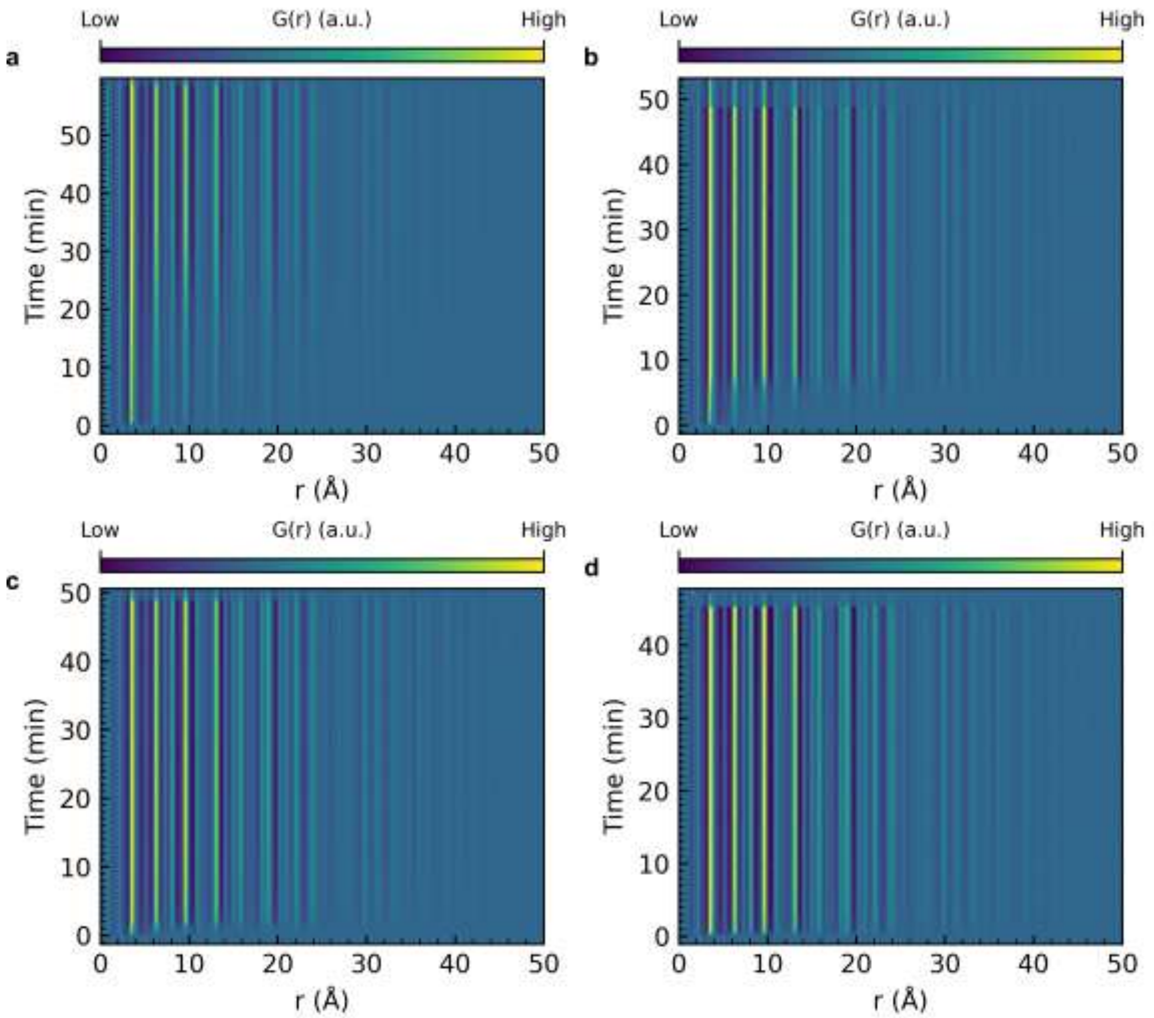

Figure S36 2D contour plot of unmodelled PDFs during the *in situ* experiments of the temperature series (x = 0.24) with synthesis temperatures of (a) 250 °C, (b) 300 °C, (c) 350 °C, and (d) 400 °C.

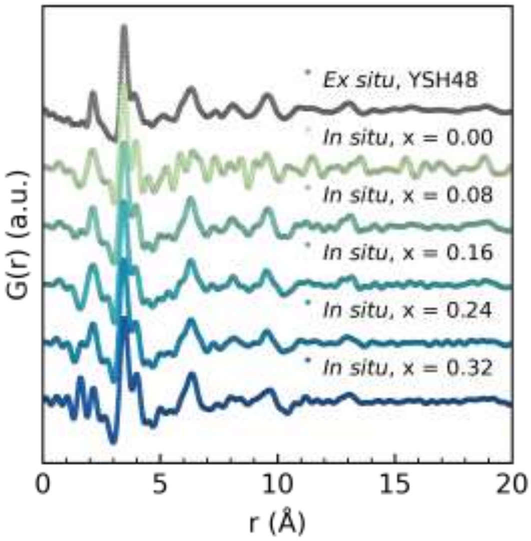

Figure S37 Comparison of PDFs of as-prepared YSH48 sample and PDFs obtained after 60 s of *in situ* synthesis experiment of all compositions. The PDF from the *in situ* experiment is obtained by summing data frames to give a five second exposure.

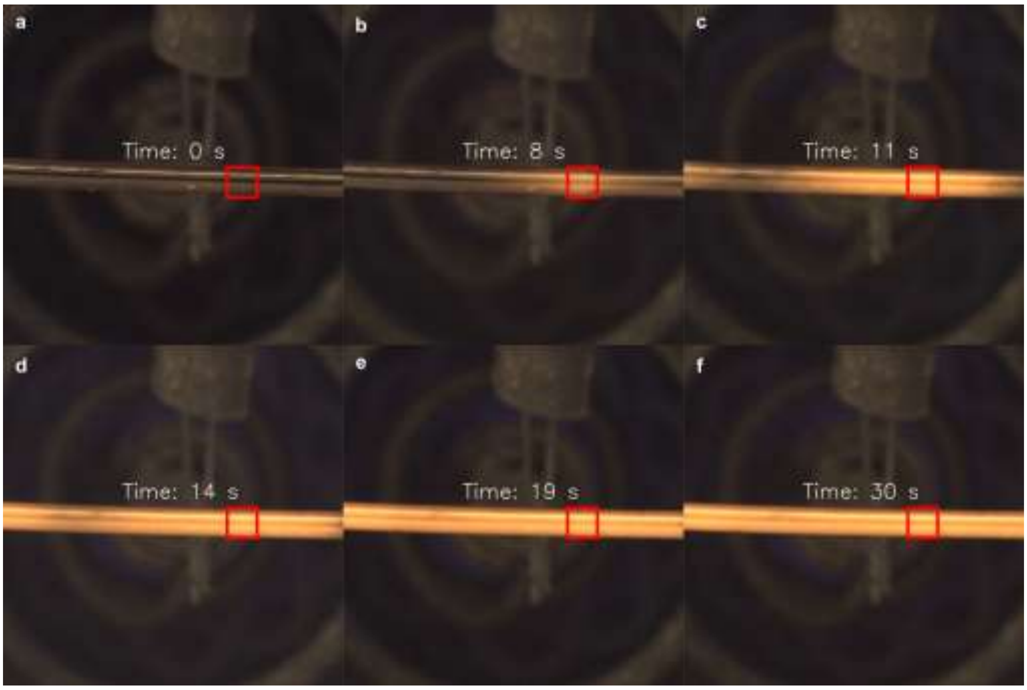

Figure S38 Photos taken during *in situ* solvothermal synthesis experiment (x = 0.24, 300 °C) with camera placed above the sample at different times after heating; (a) 0 s, (b) 8 s, (c) 11 s, (d) 14 s, (e) 19 s, and (f) 30 s. An approximate X-ray beam position is indicated with a red square. As part of the installation of the setup at the beamline, the beam position is calibrated to the hottest segment of the heater, since particles typically nucleate at the segment first. This is also the case here.

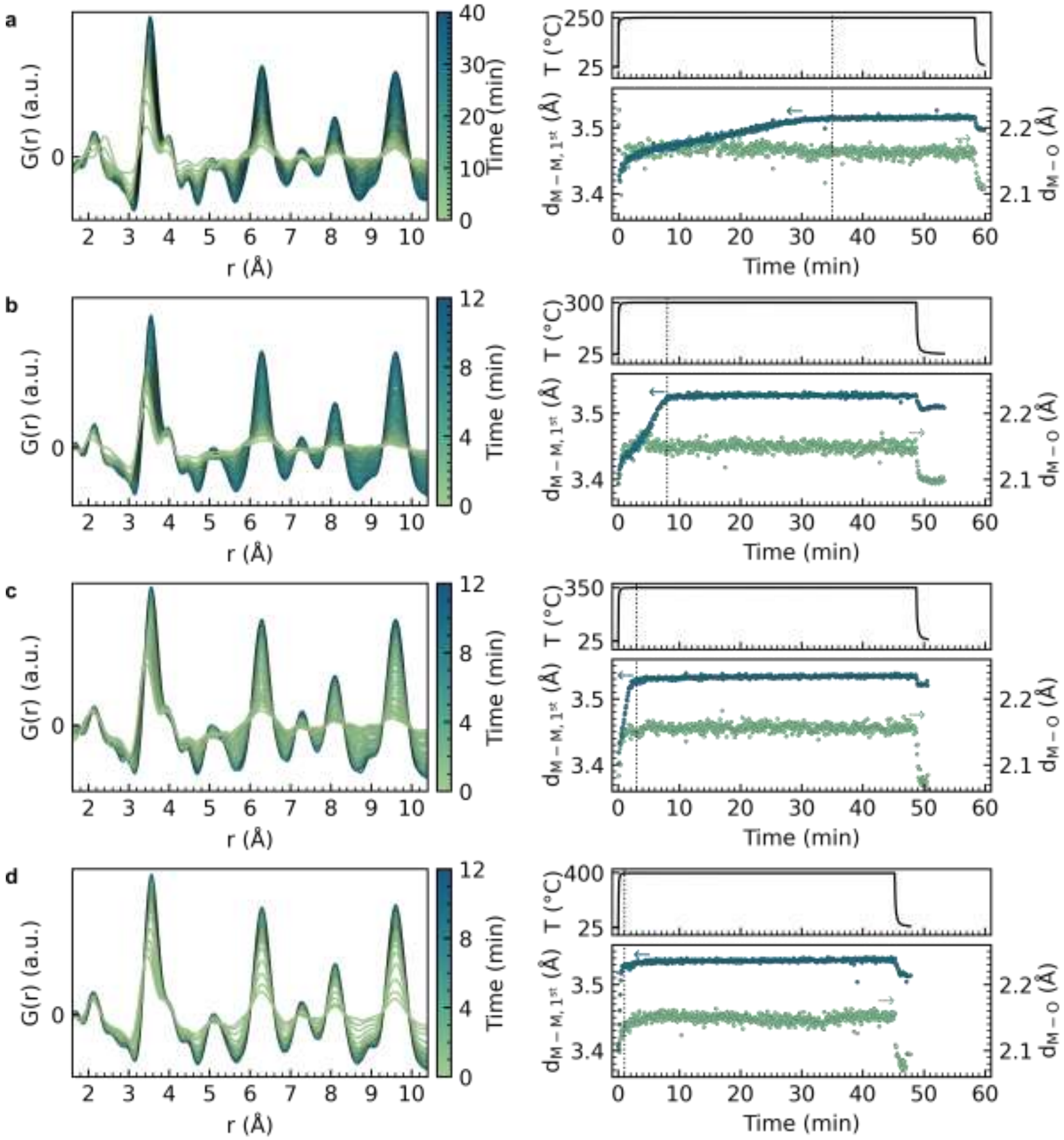

Figure S39 Low *r* region and single peak fitting of 1st M-O and 1st M-M distances of the PDFs from *in situ* experiments for x = 0.24 synthesized at (a) 250 °C, (b) 300 °C, (c) 350 °C, and (d) 400 °C. Vertical dashed line corresponds to the approximate time where the 1st M-M distance reaches a plateau.

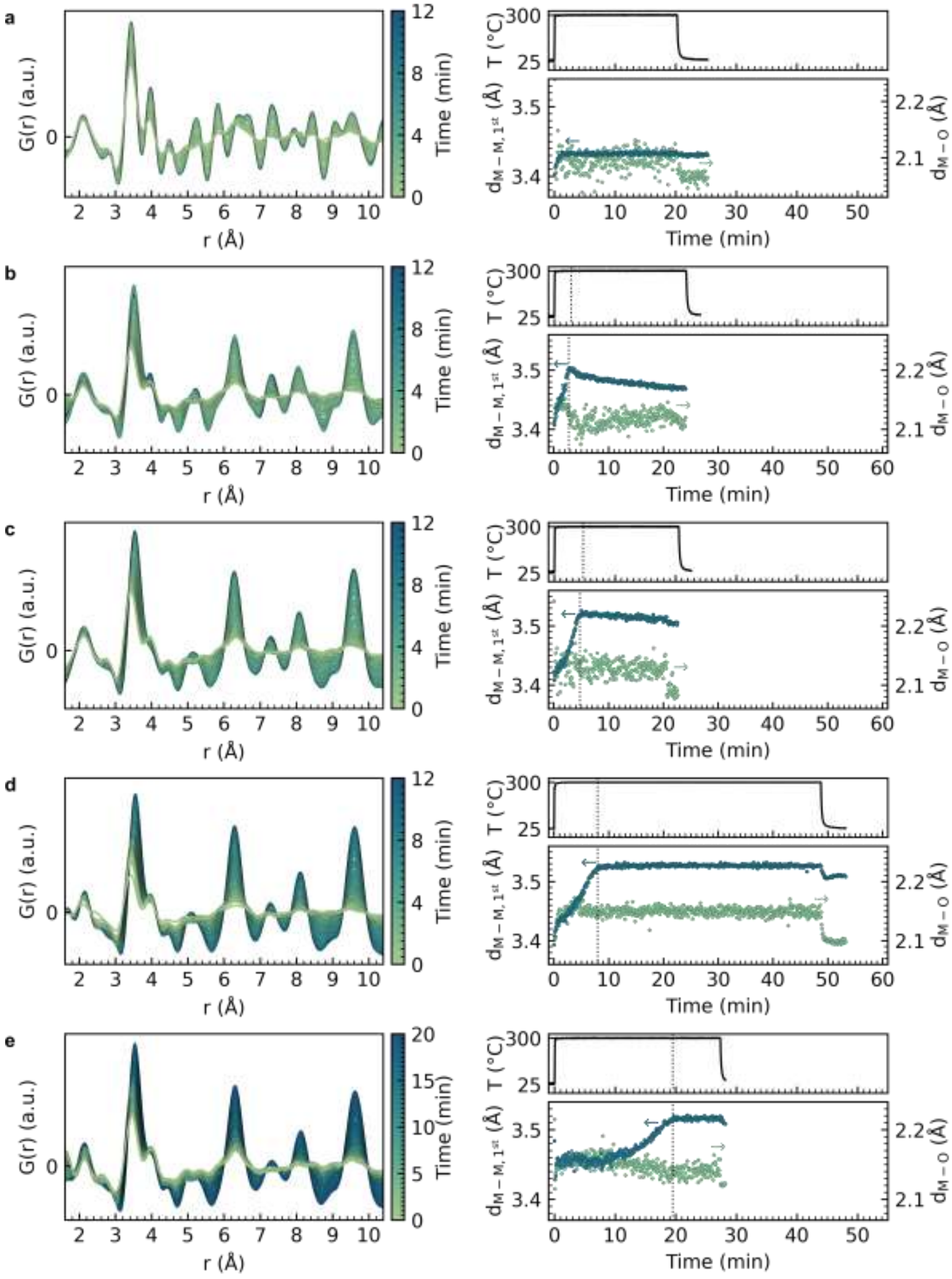

Figure S40 Low *r* region and single peak fitting of 1st M-O and 1st M-M distances of the PDFs from *in situ* experiments for (a) x = 0.00, (b) x = 0.08, (c) x = 0.16, (d) x = 0.24, and (e) x = 0.32. Vertical dashed line corresponds to the approximate time where the 1st M-
